# KDM8 acts as a co-regulator of transcription factor SOX2 for promoting cell pluripotency

**DOI:** 10.1016/j.stemcr.2026.102963

**Published:** 2026-06-18

**Authors:** Songqin Yang, Zhikai Ye, Lu Lin, Zhenlong Jiang, Erkang Wang, Jin Wang

**Affiliations:** 1 State Key Laboratory of Electroanalytical Chemistry, Changchun Institute of Applied Chemistry, Chinese Academy of Sciences, Changchun, Jilin 130022, China; 2 School of Applied Chemistry and Engineering, University of Science and Technology of China, Hefei, Anhui 230026, China; 3 Center for Theoretical Interdisciplinary Sciences Wenzhou Institute, University of Chinese Academy of Sciences, Wenzhou, Zhejiang 325001, China; 4 Department of Chemistry, Physics and Applied Mathematics, State University of New York at Stony Brook., Stony Brook, NY 11794-3400, USA

**Keywords:** KDM8, iPSCs, SOX2, co-regulator, epigenetic barrier

## Abstract

iPSCs have drawn significant attention for their biomedical potential, yet reprogramming remains inefficient and the underlying mechanisms are incompletely defined. *KDM8*, a histone demethylase, is known to play critical roles in processes such as cell-cycle regulation and embryonic development; nevertheless, its function in reprogramming has not been reported. Our investigations demonstrate that *KDM8* significantly enhances the reprogramming efficiency mediated by the canonical Yamanaka factors. Remarkably, *KDM8*, in combination with *OCT4* alone, is sufficient to reprogram somatic cells. Further analyses reveal that *KDM8* facilitates reprogramming through a dual regulatory mechanism. On one hand, KDM8 leverages its canonical enzymatic activity to reduce the epigenetic barriers to iPSC formation. More importantly, KDM8 functions as a co-regulator of the transcription factor SOX2, promoting SOX2’s DNA-binding affinity and transcriptional regulation of downstream pluripotency target genes. Accordingly, we propose a novel regulatory framework that uncovers novel mechanisms and functions of *KDM8* in cellular reprogramming.

## Highlights

• *KDM8*, in combination with *OCT4*, enables the successful generation of iPSCs
• *KDM8* reduces epigenetic barriers during reprogramming
• *KDM8* promotes reprogramming by serving as a key transcriptional co-regulator of *SOX2*
• *KDM8* is a potential pluripotency factor that possesses a dual regulatory function

## Graphical abstract

**Figure:**
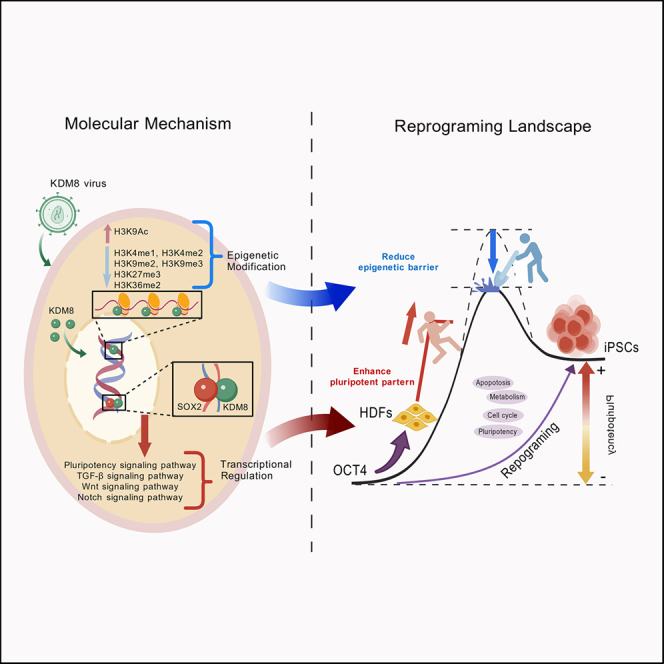

## Teaser

This study elucidates that *KDM8* promotes reprogramming through a dual mechanism: (1) enzymatically reducing the epigenetic barriers that must be overcome for iPSCs generation and (2) enhancing *SOX2* binding and transcriptional regulation of downstream gene networks.

## Introduction

Since the discovery of induced pluripotent stem cells (iPSCs), successful reprogramming has been achieved across various somatic cell lineages and multiple species (Nie et al., 2015; Takahashi and Yamanaka, 2006; Tsukamoto et al., 2024). However, the standard methodology involving transduction of the Yamanaka factors (*OCT4*, *SOX2*, *KLF4*, and *MYC*) remains a remarkably consistent yet inefficient process (Stadtfeld and Hochedlinger, 2010). This low reprogramming efficiency continues to pose significant challenges for therapeutic applications of iPSCs.

Recent research efforts have focused on elucidating molecular mechanisms of cellular reprogramming and identifying critical enhancers of this process, including *ZIC3*, *NAC1*, *PBX1*, and *GLIS1*, which have emerged as potent facilitators (Declercq et al., 2013; Faiola et al., 2017; Jiang et al., 2019; Li et al., 2020; Wang et al., 2021; Zhao et al., 2008). Our group has also actively explored this field and has previously demonstrated that the transduction-mediated overexpression of *LMCD1* or *KDM1B* can significantly enhance the efficiency of reprogramming human dermal fibroblasts (HDFs) into iPSCs (Hou et al., 2022; Ye et al., 2022). However, to date, the involvement of numerous genes in the reprogramming process remains unknown, particularly that of epigenetic factors critical for cell fate determination (Buckberry et al., 2023; Du et al., 2022).

*KDM8* (lysine demethylase 8) has been reported to catalyze H3K36me2 demethylation, modulate proliferation in both Schwann cells and cancer cells, and is critically required during the late phase of homologous recombination (HR)-mediated DNA repair to maintain genomic integrity (Fuhrmann et al., 2018; Hsia et al., 2010; Sale et al., 2017). Furthermore, KDM8 has garnered significant research interest owing to its unique JmjC (Jumonji C) domain-encoded protein hydroxylase activity, accounting for its alternative designation JMJD5 (Oh et al., 2019; Wilkins et al., 2018). Emerging evidence reveals that JMJD5 participates in regulating the reprogramming of glucose metabolism in breast cancer, and that JMJD5 deficiency underlies a syndromic human developmental disorder characterized by severe prenatal-onset growth retardation, intellectual disability, and craniofacial dysmorphism (Fletcher et al., 2023; Wang et al., 2025). *KDM8* exhibits complex biological functions, and whether it plays a role in cellular reprogramming has not been established.

To explore the role of *KDM8* in cellular reprogramming, we investigated its function and underlying mechanism. We demonstrate that augmenting the canonical OSKM reprogramming cocktail (comprising OCT4, SOX2, KLF4, and MYC) with KDM8 significantly enhances reprogramming efficiency. Notably, pluripotency reprogramming was successfully induced using only *OCT4* and *KDM8*. Furthermore, we analyzed the molecular pathways and mechanisms underlying KDM8-mediated regulation during reprogramming using western blotting (WB), co-immunoprecipitation (coIP) and chromatin immunoprecipitation (ChIP). We demonstrated that KDM8 functions not only as an epigenetic modifier that reduces epigenetic barriers during reprogramming via histone modifications but more crucially, as a co-regulator facilitating the function of the core pluripotency factor SOX2. Overall, *KDM8* promotes somatic cell reprogramming through a dual mechanism: lowering epigenetic barriers and potentiating pluripotency factor activity.

## Results

### *KDM8* promotes the generation of iPSCs

**Figure 1:**
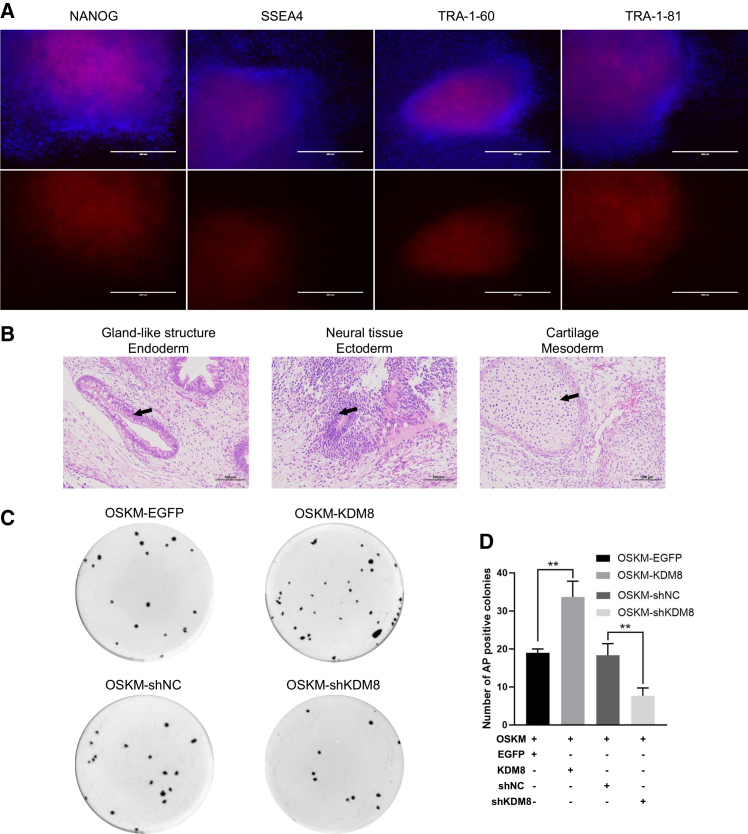
KDM8 enhances iPSCs generation (A) Immunofluorescence staining of pluripotency markers (NANOG, SSEA4, TRA-1-60, and TRA-1-81) in iPSCs-OSKM-KDM8. Single-channel and merged images (with 4',6-diamidino-2-phenylindole [DAPI] nuclear counterstain) are shown. Scale bars, 400 μm. (B) H&E staining of teratomas developed by injecting iPSCs-OSKM-KDM8 into CB-17 SCID mice, which revealed three germ layers (endoderm, mesoderm, and ectoderm) (scale bars, 100 μm). (C and D) Alkaline phosphatase positive clones of OSKM-EGFP, OSKM-KDM8, OSKM-shNC, and OSKM-shKDM8 induced HDFs into iPSCs at day 30. Data are represented as the mean ± SD, *n* = 3 independent experiments. ^∗∗^*p* < 0.01. Also, see Figure S1.

To analyze whether *KDM8* plays a functional role during cellular reprogramming, we transduced HDFs with lentiviral vectors encoding either the classic Yamanaka factors (OSKM) or OSKM plus KDM8 (OSKM-KDM8) to induce iPSCs generation. iPSCs-like colonies were harvested at day 30 post-transduction. Subsequently, these putative iPSCs colonies were subjected to rigorous pluripotency characterization. As anticipated, immunofluorescence staining confirmed that iPSCs-like colonies derived from the OSKM-KDM8 group robustly expressed key pluripotency markers, including NANOG, SSEA4, TRA-1-60, and TRA-1-81 (Figures 1A and S1A). Furthermore, upon injection into CB-17 SCID (severe combined immunodeficiency) mice, these cell clones formed teratomas containing tissues representative of all three germ layers: endoderm (glandular structures), mesoderm (cartilage), and ectoderm (neural tissue) (Figure 1B). To evaluate the impact of *KDM8* on iPSCs generation efficiency, we transduced HDFs with lentiviral vectors encoding either OSKM plus KDM8 (OSKM-KDM8) or OSKM plus a KDM8-targeting short hairpin RNA (shRNA) (OSKM-shKDM8). Alkaline phosphatase (AP) staining revealed that *KDM8* overexpression significantly increased the number of iPSCs colonies compared to the OSKM control (33.67 ± 4.16 vs. 19.33 ± 1.53 colonies; *p* < 0.01), representing an approximately 87% enhancement in reprogramming efficiency. Conversely, concomitant *KDM8* knockdown markedly reduced iPSCs colony formation (7.67 ± 2.08 vs. 18.00 ± 2.65 colonies; *p* < 0.01; Figures 1C and 1D).

### *KDM8* combined with *OCT4* can successfully complete the reprogramming process

Given the substantial enhancement of reprogramming efficiency by *KDM8*, we investigated whether *KDM8* could functionally replace individual components within the canonical Yamanaka factor (OSKM) cocktail. HDFs were transduced with lentiviral vectors encoding either the full OSKM set or ternary combinations of Yamanaka factors supplemented with KDM8 (OSK-KDM8, OSM-KDM8, OKM-KDM8, or SKM-KDM8). Notably, iPSCs-like colonies emerged not only in the OSKM control group but also in the OSK-KDM8, OSM-KDM8, and OKM-KDM8 groups (Figure S1B). Furthermore, replacing either *SOX2* or *KLF4* with *KDM8* significantly increased the number of iPSCs colonies compared to the OSKM control (25.66 ± 3.06 and 24.33 ± 2.52 colonies, respectively, vs. 19.33 ± 1.53 colonies; Figure S1C). Immunofluorescence staining confirmed that iPSCs colonies generated using *KDM8* as a substitute for *SOX2*, *KLF4*, or *MYC* robustly expressed key pluripotency markers, including NANOG, SSEA4, TRA-1-60, and TRA-1-81 (Figure S2). Furthermore, upon injection into CB-17 SCID mice, these cell clones formed teratomas containing tissues representative of all three germ layers: endoderm (glandular structures), mesoderm (osteoid-like tissue), and ectoderm (neural tissue) (Figure S1D). These results demonstrate that *KDM8* can effectively replace *SOX2*, *KLF4*, or *MYC* within the classical reprogramming cocktail, enabling successful iPSCs generation using novel four-factor combinations.

**Figure 2:**
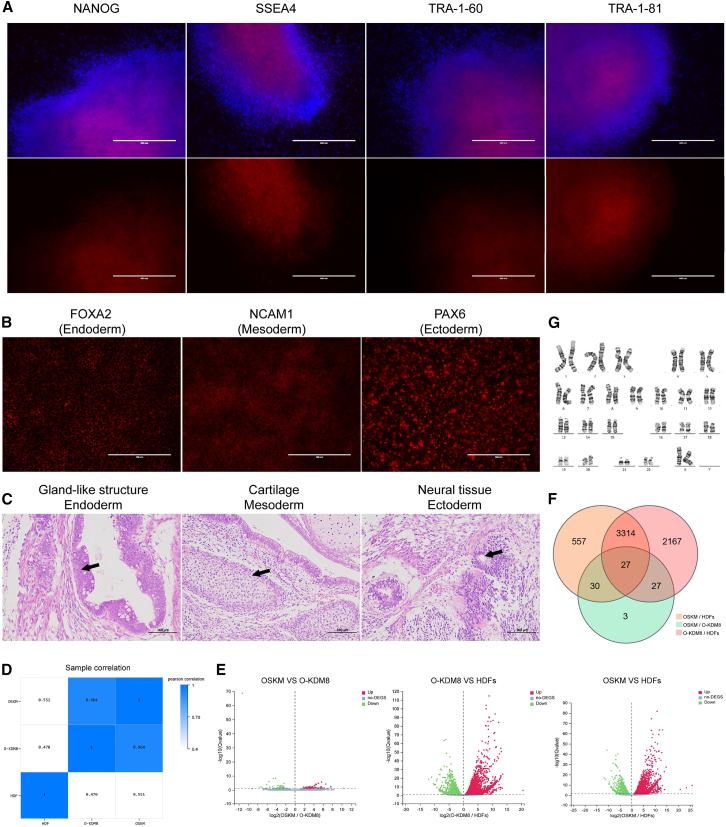
The combination of KDM8 and OCT4 can induce iPSCs (A) Immunofluorescence staining of pluripotency markers (NANOG, SSEA4, TRA-1-60, and TRA-1-81) in iPSCs-O-KDM8. Single-channel and merged images (with DAPI nuclear counterstain) are shown. Scale bars, 400 μm. (B) Immunofluorescence staining of three lineage-specific markers (PAX6, NCAM1, and FOXA2) associated with iPSCs-O-KDM8 directed differentiation to three germ layers (ectoderm, mesoderm, and endoderm) (scale bars, 400 μm). (C) H&E staining of teratomas developed by injecting iPSCs-O-KDM8 into CB-17 SCID mice, which revealed three germ layers (endoderm, mesoderm, and ectoderm) (scale bars, 100 μm). (D) RNA-seq inter-sample correlation analysis of HDFs, iPSCs-O-KDM8, and iPSCs-OSKM, Pearson’s correlation coefficient (0.964) was indicative of a correlation. (E) Volcano plots visualize differentially expressed genes (DEGs) identified by RNA-seq analysis comparing HDFs, iPSCs-O-KDM8, and iPSCs-OSKM. (F) Venn diagram of expressed genes in HDFs, iPSCs-O-KDM8, and iPSCs-OSKM based on RNA-seq data. Numbers indicate the number of expressed genes in each cell type or their intersections. (G) G-banding karyotype analysis of iPSCs-O-KDM8. The results show a normal diploid karyotype. Also see Figures S1 and S2.

Building upon the demonstrated ability of *KDM8* to enhance the reprogramming efficiency of the Yamanaka factor (OSKM) cocktail, we hypothesized that *KDM8* possesses the potential to simultaneously replace *SOX2*, *KLF4*, and *MYC* during reprogramming. To test this, we transduced HDFs with lentiviral vectors encoding only OCT4 and KDM8 (O-KDM8). As anticipated, this O-KDM8 combination successfully generated iPSCs-like colonies (iPSCs-O-KDM8) (Figures S1B and S1C). Immunofluorescence staining confirmed robust expression of key pluripotency markers (NANOG, SSEA4, TRA-1-60, and TRA-1-81) within these colonies (Figure 2A).

To further assess pluripotency, iPSCs-O-KDM8 were subjected to *in vitro* trilineage differentiation. Immunofluorescence analysis revealed efficient differentiation, with resultant cells expressing high levels of lineage-specific markers: PAX6 (ectoderm), NCAM1 (mesoderm), and FOXA2 (endoderm) (Figure 2B). Furthermore, upon injection into CB-17 SCID mice, iPSCs-O-KDM8 formed teratomas containing tissues representative of all three germ layers, including glandular epithelium (endoderm), cartilage (mesoderm), and neural rosettes (ectoderm) (Figure 2C). Comparative transcriptome analysis via RNA sequencing (RNA-seq) of uninduced HDFs, iPSCs-OSKM, and iPSCs-O-KDM8 revealed a strong positive correlation between the differential gene expression profiles of iPSCs-OSKM and iPSCs-O-KDM8 (Pearson’s correlation = 0.964, Figure 2D). Both iPSCs lines were markedly distinct from the parental HDFs (Figures 2E and 2F). Karyotype analysis confirmed that iPSCs-O-KDM8 maintained a normal chromosomal structure without detectable abnormalities (Figure 2G).

Collectively, these findings demonstrate that *KDM8* functions as a potent reprogramming enhancer capable of replacing the SKM factors (*SOX2*, *KLF4*, and *MYC*), enabling efficient iPSCs generation using only *OCT4* and *KDM8*. Notably, replacement of *SOX2* or *KLF4* with *KDM8* resulted in an appreciable increase in reprogramming efficiency, demonstrating KDM8’s capacity as a potential pluripotency factor to enhance reprogramming. However, the group where *MYC* was replaced exhibited a slight decline in reprogramming efficiency, indicating that *KDM8* cannot fully recapitulate *MYC*’s functional role in promoting reprogramming efficacy (Figure S1C). To elucidate the underlying mechanisms responsible for the remarkable efficacy of *KDM8* in reprogramming, we performed more detailed investigations.

### *KDM8* can promote cell proliferation and resist cell apoptosis

**Figure 3:**
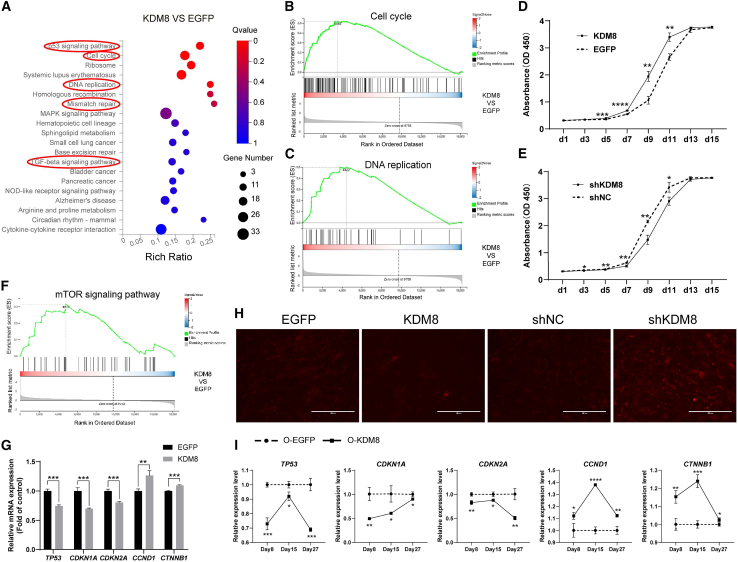
KDM8 can promote cell proliferation and resist cell apoptosis (A) KEGG pathway analysis of RNA-seq data from HDFs-KDM8 vs. HDFs-EGFP at day 5. (B and C) GSEA of RNA-seq data from HDFs-KDM8 vs. HDFs-EGFP at day 5: reactome enrichment plots showed enrichment in pathways related to cell proliferation and DNA replication. (D and E) Cell proliferation curves for HDFs expressing EGFP and KDM8 or shNC and shKDM8, which were measured by CCK-8. Data are represented as the mean ± SD, *n* = 3 independent experiments. ^∗^*p* < 0.05; ^∗∗^*p* < 0.01; ^∗∗∗^*p* < 0.001; ^∗∗∗∗^*p* < 0.0001. (F) GSEA of RNA-seq data from HDFs-KDM8 vs. HDFs-EGFP at day 5: reactome enrichment plots showed enrichment in the mTOR signaling pathway. (G) Expression of apoptosis-related genes in HDFs-KDM8 and HDFs-EGFP at day 8 was assessed by qPCR. Data are represented as the mean ± SD, *n* = 3 independent experiments. ^∗∗^*p* < 0.01; ^∗∗∗^*p* < 0.001. (H) Apoptosis induced by camptothecin in HDFs transfected with EGFP and KDM8 or shNC and shKDM8: APC represents early apoptotic cells (scale bars, 400 μm). *n* = 3 independent experiments. (I) The expression of apoptosis-related genes during the O-KDM8 reprogramming process was assessed by qPCR. Data are represented as the mean ± SD, *n* = 3 independent experiments. ^∗^*p* < 0.05; ^∗∗^*p* < 0.01; ^∗∗∗^*p* < 0.001; ^∗∗∗∗^*p* < 0.0001. Also see Figure S3.

*KDM8* has been implicated in promoting cancer cell proliferation and conferring anti-apoptotic properties (Huang et al., 2015). To investigate whether *KDM8* exerts similar effects on HDFs, we performed RNA-seq to profile global gene expression patterns in HDFs expressing either *EGFP* (control) or *KDM8*. Kyoto Encyclopedia of Genes and Genomes (KEGG) pathway enrichment analysis (Figure 3A; Table S1) and gene set enrichment analysis (GSEA) (Figures 3B and 3C) revealed significant enrichment of differentially expressed genes in pathways related to cell proliferation and DNA repair in KDM8-expressing cells compared to controls, indicating that *KDM8* modulates these signaling cascades.

We next directly assessed the impact of *KDM8* on cell proliferation, comparing cells overexpressing *KDM8* (HDFs-KDM8) versus cells overexpressing *EGFP* (HDFs-EGFP) (Figure S3A), and cells overexpressing shKDM8 (HDFs-shKDM8) versus cells overexpressing negative control shRNA (HDFs-shNC) (Figure S3B). Cell Counting Kit-8 (CCK-8) assays demonstrated that exogenous *KDM8* expression significantly enhanced HDFs proliferation relative to its respective control (Figure 3D), whereas *KDM8* knockdown via shRNA markedly reduced HDFs growth rates (Figure 3E). Cell cycle analysis further revealed that *KDM8* overexpression promoted cell cycle progression, shifting HDFs from G0/G1 phase into S phase (Figures S3C and S3D). Consequently, the proliferation index (PI) was significantly elevated (*p* < 0.0001; Figure S3E). Conversely, *KDM8* knockdown resulted in a higher proportion of HDFs arrested in G0/G1 phase and a significant reduction in PI (*p* < 0.01; Figures S3F–S3H). These results demonstrate that *KDM8* effectively regulates the cell cycle and promotes proliferation in HDFs.

GSEA revealed significant enrichment of the mTOR signaling pathway in KDM8-overexpressing cells (Figure 3F). Subsequent qPCR analysis of HDFs-KDM8 demonstrated that *KDM8* upregulation significantly increased the expression levels of *CCND1* (*p* < 0.01) and *CTNNB1* (*p* < 0.001), while decreasing transcript levels of *TP53* (*p* < 0.001), *CDKN1A* (*p* < 0.001), and *CDKN2A* (*p* < 0.001) (Figure 3G). This indicates that *KDM8* overexpression suppresses key pro-apoptotic regulators. Additionally, to directly assess anti-apoptotic capacity, cells were treated with camptothecin to induce apoptosis and stained with Annexin-V-Allophycocyanin (APC) to detect early apoptotic cells. Qualitative assessment revealed fewer Annexin-V-APC-positive (early apoptotic) cells in the KDM8-overexpression group compared to controls, whereas *KDM8* knockdown increased early apoptosis (Figure 3H). More rigorously, dual staining with Annexin-V-APC and 7-AAD followed by flow cytometric analysis quantified apoptotic populations. *KDM8* overexpression significantly reduced the proportion of both early apoptotic (Annexin V^+^/7-AAD^-^; *p* < 0.01) and late apoptotic (Annexin V^+^/7-AAD^+^; *p* < 0.001) cells (Figures S3I–S3K). Conversely, *KDM8* knockdown significantly increased early (*p* < 0.0001) and late apoptotic (*p* < 0.05) populations (Figures S3L–S3N). These results demonstrate that *KDM8* overexpression confers enhanced resistance to apoptosis. Furthermore, during iPSCs generation using the *OCT4* combined *KDM8* reprogramming system, we observed concomitant downregulation of apoptosis-related genes and upregulation of proliferation-associated genes, providing additional corroboration for the above findings (Figure 3I).

### *KDM8* promotes the TGF-β signaling pathway and cellular glycolytic metabolism

**Figure 4:**
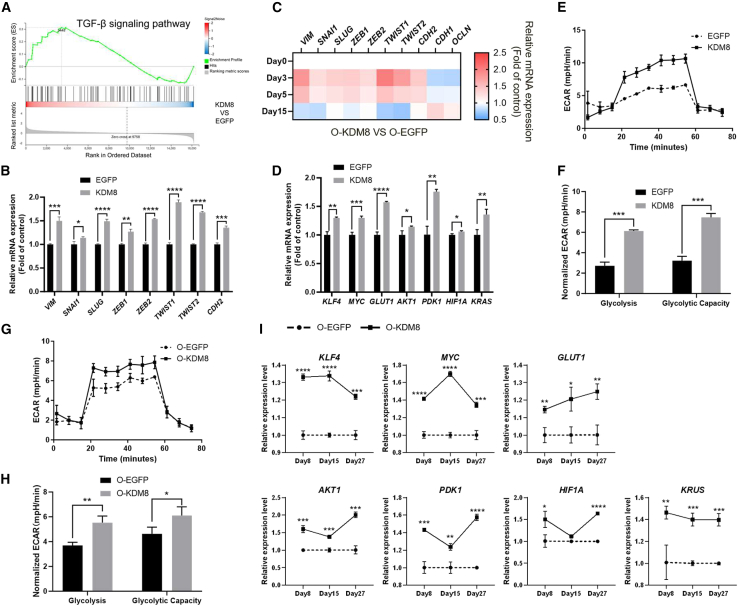
KDM8 promotes the TGF-β signaling pathway and glycolytic metabolism (A) GSEA of RNA-seq data from HDFs-KDM8 vs. HDFs-EGFP at day 5: reactome enrichment plots showed enrichment in the TGF-β signaling pathway. (B) Expression of TGF-β signaling pathway related genes in HDFs-KDM8 and HDFs-EGFP at day 5 was assessed by qPCR. Data are represented as the mean ± SD, *n* = 3 independent experiments. ^∗^*p* < 0.05; ^∗∗^*p* < 0.01; ^∗∗∗^*p* < 0.001; ^∗∗∗∗^*p* < 0.0001. (C) Expression of TGF-β signaling pathway genes was quantified by qPCR in HDFs-O-KDM8 vs. HDFs-O-EGFP at days 3, 5, and 15 post-transduction. Data are represented as the mean ± SD, *n* = 3 independent experiments. (D) The expression of cell metabolism related genes in HDFs-KDM8 and HDFs-EGFP at day 8 was assessed by qPCR. Data are represented as the mean ± SD, *n* = 3 independent experiments. ^∗^*p* < 0.05; ^∗∗^*p* < 0.01; ^∗∗∗^*p* < 0.001; ^∗∗∗∗^*p* < 0.0001. (E and F) Glycolysis function in HDFs-KDM8 and HDFs-EGFP at day 5. Data are represented as the mean ± SD, *n* = 3 independent experiments. ^∗∗∗^*p* < 0.001. (G and H) Glycolysis function in HDFs-O-KDM8 and HDFs-O-EGFP at day 5. Data are represented as the mean ± SD, *n* = 3 independent experiments. ^∗^*p* < 0.05; ^∗∗^*p* < 0.01. (I) The expression of cell metabolism related genes during the O-KDM8 reprogramming process was assessed by qPCR. Data are represented as the mean ± SD, *n* = 3 independent experiments. ^∗^*p* < 0.05; ^∗∗^*p* < 0.01; ^∗∗∗^*p* < 0.001; ^∗∗∗∗^*p* < 0.0001. Also see Figure S4.

Both KEGG pathway and GSEA indicated that *KDM8* modulates the TGF-β signaling pathway (Figures 3A and 4A). To characterize this regulatory relationship, we quantified expression levels of TGF-β pathway-associated genes via qPCR. On day 5 post-KDM8 overexpression, significant upregulation was observed for *VIM* (*p* < 0.001), *SNAI1* (*p* < 0.05), *SNAI2* (*p* < 0.0001), *ZEB1* (*p* < 0.01), *ZEB2* (*p* < 0.0001), *TWIST1* (*p* < 0.0001), *TWIST2* (*p* < 0.0001), and *CDH2* (*p* < 0.001) (Figure 4B). By contrast, transcript levels of these genes returned to baseline by day 15 (Figure S4A). Given this phenomenon, we examined the expression levels of TGF-β pathway-related genes during the early reprogramming phase in HDFs-O-KDM8. qPCR results revealed that epithelial-mesenchymal transition (EMT)-associated genes (*VIM*, *SNAI1*, *SLUG*, *ZEB1*, *ZEB2*, *TWIST1*, *TWIST2*, and *CDH2*) were upregulated on days 3 and 5, while mesenchymal-epithelial transition (MET)-associated genes (*CDH1* and *OCLN*) were suppressed. By day 15, EMT genes were downregulated, and MET genes were activated (Figure 4C). This temporal pattern demonstrates that *KDM8* overexpression induces upregulation of TGF-β-responsive genes during early reprogramming stages, an effect that dissipates in later phases.

Enhanced glycolysis is generally considered to be advantageous for reprogramming, particularly during its early phases (Ishida et al., 2020). Given that cellular reprogramming involves metabolic remodeling, we assessed the impact of *KDM8* overexpression on metabolic gene expression in HDFs via qPCR (Jia et al., 2021; Sun et al., 2020). As shown in Figure 4D, *KDM8* overexpression significantly upregulated transcripts encoding *KLF4* (*p* < 0.01), *MYC* (*p* < 0.001), *GLUT1* (*p* < 0.0001), *AKT1* (*p* < 0.05), *PDK1* (*p* < 0.01), *HIF1A* (*p* < 0.05), and *KRAS* (*p* < 0.01). Furthermore, Seahorse metabolic flux analysis confirmed KDM8-mediated enhancement of glycolytic function, as measured by extracellular acidification rate (ECAR). Specifically, *KDM8* overexpression significantly elevated both basal glycolysis and glycolytic capacity in HDFs (Figures 4E and 4F), whereas *KDM8* knockdown suppressed these parameters (Figures S4B–S4E). Consistent with these findings, HDFs-O-KDM8 exhibited enhanced basal glycolysis and glycolytic capacity compared to HDFs-O-EGFP controls (Figures 4G and 4H), whereas no significant differences in mitochondrial oxidative phosphorylation levels at the same time (Figures S4F and S4G). Concurrently, metabolic genes related to glycolytic metabolism were upregulated during iPSC generation using the O-KDM8 reprogramming system (Figure 4I). These results further supports a metabolic reprogramming-modulating role for *KDM8*.

### *KDM8* can function as a co-regulator of *SOX2* in its transcriptional regulation

Collectively, these experiments demonstrate that *KDM8* enhances cell proliferation, confers apoptosis resistance, regulates metabolic functions, and modulates TGF-β signaling pathways. These functional attributes are associated with somatic cell reprogramming competence, as referenced in prior studies (Xu et al., 2016). However, the mechanistic basis underlying the remarkable ability of *KDM8* to functionally substitute for *SOX2*, *KLF4*, and *MYC* concurrently remains incompletely understood, warranting deeper investigation into its regulatory mechanisms.

**Figure 5:**
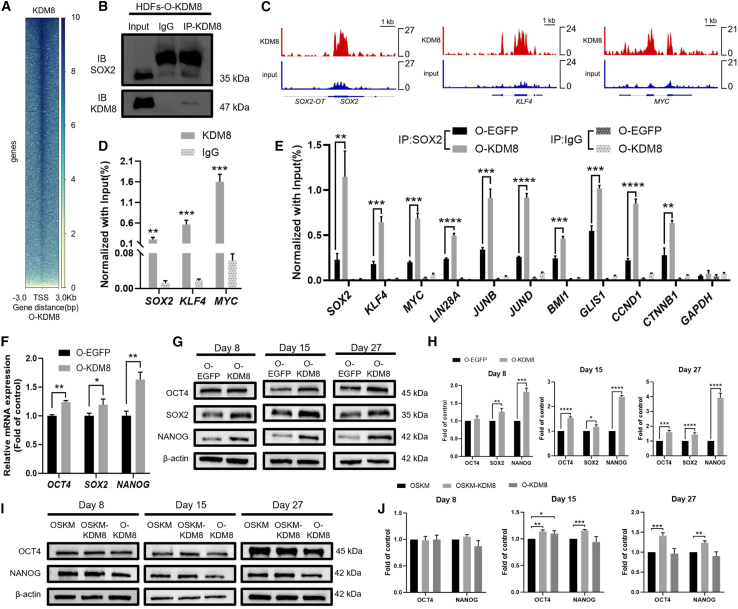
KDM8 binds to SOX2 to regulate gene transcription (A) A heatmap of KDM8 ChIP-seq occupancy around the TSS (±3 kb) in HDFs-O-KDM8 at day 8. (B) The protein interaction between KDM8 and SOX2 in HDFs-O-KDM8 was assessed by coIP at day 8. (C) Genome views of KDM8 tag density at *SOX2*, *KLF4*, and *MYC* in HDFs-O-KDM8. (D) ChIP-qPCR analysis of KDM8 occupancy at *SOX2*, *KLF4*, and *MYC* loci in HDFs-O-KDM8 at day 8. Data are represented as the mean ± SD, *n* = 3 independent experiments. ^∗∗^*p* < 0.01; ^∗∗∗^*p* < 0.001. (E) ChIP-qPCR analysis of SOX2 occupancy at reprogramming-associated loci in HDFs-O-KDM8 and HDFs-O-EGFP. Data are represented as the mean ± SD, *n* = 3 independent experiments. ^∗∗^*p* < 0.01; ^∗∗∗^*p* < 0.001; ^∗∗∗∗^*p* < 0.0001. (F) Expression of *OCT4*, *SOX2* and *NANOG* for HDFs-O-KDM8 and HDFs-O-EGFP was assessed by qPCR at day 8. Data are represented as the mean ± SD, *n* = 3 independent experiments. ^∗^*p* < 0.05; ^∗∗^*p* < 0.01. (G and H) Western blot analysis of the levels of OCT4, SOX2, and NANOG proteins in cells transduced by O-KDM8 and O-EGFP at day 8, 15, and 27. β-Actin was used as an endogenous control for equal loading. Data are represented as the mean ± SD, *n* = 3 independent experiments. ^∗^*p* < 0.05; ^∗∗^*p* < 0.01; ^∗∗∗^*p* < 0.001; ^∗∗∗∗^*p* < 0.0001. (I and J) Western blot analysis of the levels of OCT4 and NANOG proteins in cells transduced by OSKM, OSKM-KDM8, and O-KDM8 at day 8, 15, and 27. β-Actin was used as an endogenous control for equal loading. Data are represented as the mean ± SD, *n* = 3 independent experiments. ^∗^*p* < 0.05; ^∗∗^*p* < 0.01; ^∗∗∗^*p* < 0.001. Also see Figure S5.

We performed KDM8-targeted ChIP sequencing (ChIP-seq) on HDFs expressing *OCT4* and *KDM8* (HDFs-O-KDM8). Intriguingly, heatmap analysis revealed significant region-specific enrichment of KDM8 at promoter-proximal genomic loci (Figures 5A and S5A). This suggests a potential role for KDM8 in transcriptional regulation, despite its conventional classification as a histone demethylase expected to exhibit diffuse genomic localization rather than discrete enrichment patterns. Consequently, we hypothesized that KDM8 may exert genome-wide transcriptional control through interactions with transcription factors.

Prior studies have established that SOX2, a core pluripotency transcription factor, regulates the expression of genes critical for pluripotency, including *KLF4* and *MYC* (Park et al., 2011; Tao et al., 2012; Xie et al., 2017). Building upon reported evidence of a putative KDM8-SOX2 interaction in brain endothelial cells (Yao et al., 2019), we postulated that the observed promoter enrichment of KDM8 may reflect its novel regulatory relationship with this key pluripotency factor. To test this hypothesis, we performed coIP coupled with mass spectrometry (MS), revealing SOX2-KDM8 interaction (Table S2). Subsequent western blot analysis further confirmed this association (Figure 5B). Moreover, upon *SOX2* knockdown, KDM8 enrichment at the promoter region was markedly reduced, suggesting that KDM8 is recruited to promoters through its interaction with SOX2, where it accumulates at high levels (Figures S5B–S5D).

To further investigate whether KDM8 regulates downstream gene transcription, we performed KDM8-targeted ChIP in HDFs-O-KDM8. Intriguingly, both ChIP-seq and subsequent ChIP-qPCR validation assays demonstrated direct genomic binding of KDM8 to regulatory regions of *SOX2*, *KLF4*, and *MYC* (Figures 5C and 5D). To directly validate the transcriptional regulatory function of KDM8, we performed SOX2-targeted ChIP in HDFs-O-KDM8, using HDFs-O-EGFP as controls. Heatmap analysis, together with representative ChIP-seq tracks, revealed enhanced SOX2 enrichment at promoter-proximal loci in HDFs-O-KDM8 compared to HDFs-O-EGFP (Figures S5E and S5F). ChIP-qPCR quantification confirmed significant enhancement of SOX2 occupancy at previously reported reprogramming-associated gene loci (Boyer et al., 2005; Hagey et al., 2022; Niharika et al., 2024) in HDFs-O-KDM8 relative to HDFs-O-EGFP controls. Specifically, binding was enriched at the *SOX2* autoregulatory site (*p* < 0.01), as well as at *KLF4* (*p* < 0.001), *MYC* (*p* < 0.001), *LIN28A* (*p* < 0.0001), *JUNB* (*p* < 0.001), *JUND* (*p* < 0.0001), *BMI1* (*p* < 0.001), *GLIS1* (*p* < 0.001), *CCND1* (*p* < 0.0001), and *CTNNB1* (*p* < 0.01) regulatory elements (Figure 5E). This indicates that KDM8, through its interaction with SOX2, enhances SOX2-mediated transcriptional regulation of these pluripotency factors. Furthermore, KDM8 ChIP-seq in HDFs-O-KDM8 identified more SOX2 target genes (Table S3). RNA-seq analysis of HDFs overexpressing *KDM8* showed that these genes exhibited subtle but consistent shifts favorable for reprogramming compared with the control group (Figures S5G and S5H), providing corroborating evidence that KDM8 functions as a transcriptional co-regulator of SOX2-mediated transcriptional activity.

We also investigated the impact of *KDM8* on core pluripotency circuitry. qPCR analysis showed *KDM8* upregulation significantly increased transcript levels of *OCT4* (*p* < 0.01), *SOX2* (*p* < 0.05), and *NANOG* (*p* < 0.01) (Figure 5F). Western blotting confirmed corresponding protein-level elevations at multiple time points (Figures 5G and 5H). Given that cells transfected with *OCT4* and *KDM8* progress to iPSCs while controls (cells transfected with *OCT4* and *EGFP*) do not, we systematically compared protein dynamics of core pluripotency factors across different reprogramming cocktails (OSKM, OSKM-KDM8, and O-KDM8) at key time points (Figures 5I and 5J). Notably, the OSKM-KDM8 group exhibited enhanced OCT4 and NANOG expression relative to OSKM alone, while the OCT4-KDM8 combination achieved protein levels comparable to the canonical OSKM cocktail. This demonstrates that *KDM8* not only amplifies core pluripotency factor expression but also functionally compensates for the absence of *SOX2*, *KLF4*, and *MYC*.

### *KDM8* can modify various H3 histones

**Figure 6:**
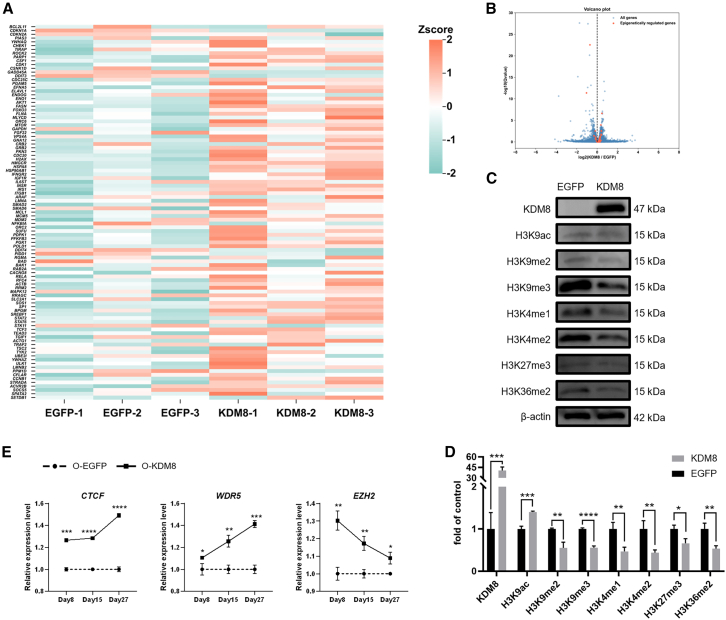
KDM8 affects multiple histone modifications (A) Heatmap of genes regulated by KDM8 through epigenetic modification plotted using RNA-seq data of HDFs-KDM8 vs. HDFs-EGFP at day 5. (B) Volcano plot of RNA-seq data (HDFs-KDM8 vs. HDFs-EGFP, day 5). Genes presented in the heatmap (Figure 6A) are highlighted. (C and D) Western blot analysis of the expression levels of KDM8, H3K9ac, H3K9me2, H3K9me3, H3K4me1, H3K4me2, H3K27me3, and H3K36me2 proteins in HDFs expressing EGFP and KDM8 at day 8. β-Actin was used as an endogenous control for equal loading. Data are represented as the mean ± SD, *n* = 3 independent experiments. ^∗^*p* < 0.05; ^∗∗^*p* < 0.01; ^∗∗∗^*p* < 0.001; ^∗∗∗∗^*p* < 0.0001. (E) The expression of *CTCF*, *WDR5*, and *EZH2* during the O-KDM8 reprogramming process was assessed by qPCR. Data are represented as the mean ± SD, *n* = 3 independent experiments. ^∗^*p* < 0.05; ^∗∗^*p* < 0.01; ^∗∗∗^*p* < 0.001; ^∗∗∗∗^*p* < 0.0001.

ChIP-seq profiling of KDM8 in HDFs-O-KDM8 cells identified additional KDM8-occupied loci not annotated as SOX2 targets (Table S4), with associated genes involved in cell proliferation, pluripotency, and differentiation. RNA-seq analysis of HDFs overexpressing KDM8 showed altered expression of these non-canonical targets. (Figures 6A and 6B). We hypothesized this reflects the canonical enzymatic function of KDM8 in histone modification-mediated gene regulation (Shen et al., 2017).

To test this, we infected HDFs with lentiviral vectors encoding *KDM8* or *EGFP* control. Western blot analysis confirmed successful KDM8 overexpression at the protein level. Notably, KDM8 overexpression significantly reduced global levels of H3K4me1 (*p* < 0.01), H3K4me2 (*p* < 0.01), H3K9me2 (*p* < 0.01), H3K9me3 (*p* < 0.0001), H3K27me3 (*p* < 0.05), and H3K36me2 (*p* < 0.01), while concurrently elevating H3K9ac levels (*p* < 0.001) (Figures 6C and 6D). Furthermore, during iPSCs generation using the O-KDM8 reprogramming system, we observed concomitant upregulation of key epigenetic regulators including *CTCF*, *WDR5*, and *EZH2* (Figure 6E). These findings collectively demonstrate the capacity of KDM8 to orchestrate epigenetic remodeling through its enzymatic functions.

## Discussion

iPSCs exhibit pluripotency comparable to embryonic stem cells (ESCs), enabling differentiation into virtually all somatic cell types (Takahashi et al., 2007). This confers significant potential for applications in regenerative medicine, disease modeling, and drug development (Abbott, 2024; Götz and Torres-Padilla, 2025). However, conventional iPSC reprogramming remains limited by inefficient multi-factor genomic integration and poorly understood molecular mechanisms. Consequently, identifying enhanced reprogramming factors and elucidating the mechanistic underpinnings of reprogramming continue to represent critical goals in iPSC research.

Prior studies indicate that KDM8, functioning as a demethylase with putative hydroxylase activity, plays multifaceted roles in embryonic development and cellular proliferation (Fletcher et al., 2023). This functional complexity has hindered comprehensive understanding of the regulatory mechanisms of *KDM8*, leaving its function in cellular reprogramming largely unexplored.

Our findings demonstrate that *KDM8* substantially enhances reprogramming efficiency mediated by the canonical Yamanaka factors. AP staining revealed the increase in iPSCs colony formation upon *KDM8* co-expression (Figures 1C and 1D). Remarkably, *KDM8* functionally substitutes for the SKM triad , enabling successful reprogramming with only *OCT4* and *KDM8* (Figure S1B). Transcriptomic profiling (Figures 2D–2F) and teratoma formation assays (Figure 2C) confirmed that O-KDM8 derived iPSCs (iPSCs-O-KDM8) exhibit gene expression profiles highly concordant with iPSCs-OSKM and possess multilineage differentiation capacity akin to ESCs. While the data indicate that replacing *MYC* with *KDM8* reduces cellular reprogramming efficiency (Figures S1B and S1C), suggesting *KDM8* cannot fully substitute *MYC*’s function, we hypothesize that this discrepancy arises from differences in their functional capacities. While both *MYC* and *KDM8* enhance cell proliferation, anti-apoptosis and glycolytic metabolism (Figures 3 and 4), *KDM8* exhibits weaker efficacy compared to *MYC*, thereby failing to achieve complete functional replacement.

RNA-seq analysis revealed that *KDM8* overexpression in HDFs impacts pathways including cell cycle progression (Figure 3B), DNA replication (Figure 3C), and mTOR signaling (Figure 3F). Concomitantly, *KDM8* enhances: proliferative capacity (Figures S3C–S3E), anti-apoptotic competence (Figures 3H and S3I–S3K), and glycolytic metabolism (Figures 4E and 4F), which are considered to be conducive to reprogramming (Guo et al., 2025; Xu et al., 2016). Notably, *KDM8* overexpression instigates time-delimited activation of TGF-β-responsive gene expression during early reprogramming stages, an effect that abates in later phases (Figures 4B and 4C). This transient induction parallels the documented EMT to MET progression during reprogramming, where early stage EMT enhances cellular plasticity and boosts initial reprogramming efficiency (Jia et al., 2019; Sun et al., 2020; Xing and Tian, 2019). Consistent with prior reports (Hsia et al., 2010), KDM8 functions as an H3K36me2-demethylating enzyme (Figures 6C and 6D). Recent evidence demonstrates that H3K36me2 depletion promotes MET while suppressing TGF-β target gene expression (Hoetker et al., 2023). This may mechanistically explain the observed downregulation of EMT-related genes in HDFs-O-KDM8 at day 15 (Figure 4C), as the regulatory effects of H3K36me2 modulation require temporal accumulation to manifest fully.

An intriguing observation emerged from KEGG enrichment analysis and GSEA: compared with the EGFP group, KDM8-overexpressing cells showed significant enrichment of Wnt signaling (Figure S6A), a pathway known to support pluripotency acquisition (Liu et al., 2021). This suggests *KDM8* may facilitate reprogramming through activation of the Wnt signaling pathway. Given that *OCT4* is a core pluripotency factor with strong regulatory effects on Wnt and related pluripotency pathways (Abu-Remaileh et al., 2010; van den Berg et al., 2010), we anticipated that introducing *OCT4* (O-KDM8 vs. O-EGFP) would diminish the apparent enrichment of differentially expressed genes in pluripotency-related pathways relative to KDM8-versus-EGFP contrast, thereby masking KDM8-specific effects (Figure S6B). Unexpectedly, although co-expression of *OCT4* and *KDM8* did attenuate the enrichment of differentially expressed genes in cell-cycle regulation, DNA replication and TGF-β signaling when comparing O-KDM8 with O-EGFP relative to the KDM8-versus-EGFP comparison (Figures 3A and S6C–S6F; Table S5), the introduction of *OCT4* instead potentiated pluripotency-associated signaling: Wnt signaling (Figure S6G) remained enriched, whereas Notch signaling (Figure S6H) and other pluripotency-associated pathways (Figure S6I) exhibited *de novo* enrichment.

In order to explain this interesting phenomenon and define how *KDM8* orchestrates cellular functions while functionally compensating for the SKM reprogramming triad, we conducted a series of more detailed analyses. Surprisingly, ChIP-seq analysis revealed significant enrichment of KDM8 at promoter regions across the genome (Figures 5A and S5A), suggesting a prominent role in transcriptional regulation. This finding attracted our attention, as histone-modifying enzymes typically exhibit more uniform genomic distribution. Critically, coIP assays confirmed direct physical interaction between KDM8 and the pluripotency factor SOX2 (Figure 5B). Consistent with this, ChIP-seq analysis demonstrated that *SOX2* knockout markedly attenuated KDM8 enrichment at promoter regions (Figures S5B–S5D). These results, combined with integrated analysis of KDM8 chromatin occupancy at canonical SOX2 targets (ChIP-seq; Table S3) and corresponding expression profiles in HDFs-KDM8 (RNA-seq; Figures S5G and S5H) confirms KDM8’s essential function as a transcriptional co-regulator in somatic cell reprogramming.

More directly, ChIP assays confirmed KDM8 binding to the *SOX2*, *KLF4*, and *MYC* genes (Figure 5D). ChIP signals for SOX2 protein at reprogramming-associated genomic loci were enhanced following KDM8 overexpression (Figures 5E, S5E, and S5F). Furthermore, ChIP-seq analysis confirmed KDM8 binding to the *BMI1*, *GATA2*, *BMP6*, and *GLIS1* genes (Table S3), which are established SOX2 targets and functionally substitute for core reprogramming factors in pluripotency induction (King and Klose, 2017; Xiao et al., 2016). This finding partially accounts for the potent capacity of *KDM8* to functionally replace *SOX2*, *KLF4*, and *MYC* during reprogramming. Moreover, as a transcriptional co-regulator, KDM8 also binds to genes associated with the TGF-β, Wnt, and Notch signaling pathways, established regulators of cellular proliferation, metabolic reprogramming, and somatic cell reprogramming competence (Niharika et al., 2024) (Table S3). These data provide evidence supporting the association between *KDM8* overexpression and phenotypic alterations, including regulation of TGF-β signaling, enhanced proliferation, apoptosis resistance, and accelerated glycolytic flux. (Figures 3 and 4). Integrating these findings, we demonstrate that *OCT4* co-expression with *KDM8* potentiates inter-group divergence within the pluripotency pathway and significantly elevates core reprogramming factors (*SOX2*, *KLF4*, and *MYC*), which suggest a mechanism that contributes to the efficacy of O-KDM8 system in inducing iPSCs.

Furthermore, ChIP-seq analysis revealed that KDM8 also bound to numerous genes not annotated as SOX2 targets (Table S4), which were modulated in KDM8-overexpressing HDFs based on RNA-seq data (Figures 6A and 6B). Given the pervasive regulatory influence of histone modifications on gene expression (Hsia et al., 2010; Shen et al., 2017), we propose that KDM8 modulates these targets via its intrinsic catalytic demethylase activity. Western blot analysis (Figures 6C and 6D) revealed that KDM8 overexpression alters histone modification patterns, including reduced levels of H3K4me1, H3K4me2, H3K9me2, H3K9me3, H3K27me3, and H3K36me2, along with increased H3K9ac enrichment, which are considered to alleviate epigenetic barriers. (Bruno Di et al., 2016; Hou et al., 2022; Liang and Zhang, 2012). Notably, in addition to the known regulation of H3K36me2 (Sale et al., 2017), we observed changes at histone modification sites not previously reported as KDM8 targets. These effects extend beyond the known histone-modifying functions of KDM8 and are likely the result of indirect consequences of its catalytic activity. Consistent with this, key epigenetic regulators (*CTCF*, *WDR5*, and *EZH2*) were coordinately upregulated during iPSC generation using the O-KDM8 reprogramming system (Figure 6E). These findings demonstrate that *KDM8* modulates cell reprogramming by orchestrating epigenetic remodeling through suppression of barrier-associated histone modifications and activation of pluripotency-linked epigenetic marks. We consequently posit that KDM8 promotes reprogramming through both assisting SOX2-driven transcriptional regulation and reducing epigenetic obstacles during the process.

To further validate the functions of KDM8 in reprogramming, we engineered complementary mutants targeting distinct functional domains. SOX2-binding defective mutant (KDM8^E301–304A^) generated by alanine substitution of the C-terminal acidic cluster (EEEE^301-304^→AAAA) to disrupt the SOX2 interaction interface (Figures S7A–S7D). Catalytically inactive mutant (KDM8^CD^) created via mutation of Fe^2+^-coordinating residues in the JmjC domain (D213A/H214A/H276A) to ablate demethylase activity (Figures S7E–S7H). When co-expressed with OCT4 during reprogramming, KDM8^E301–304A^ induced a significant reduction in iPSC generation efficiency (*p* < 0.01), whereas KDM8^CD^ exhibited a moderate inhibitory effect (*p* < 0.05) (Figures S7I and S7J). These findings suggest that KDM8 facilitates reprogramming through both assisting SOX2-driven transcriptional regulation and reducing epigenetic obstacles.

**Figure 7:**
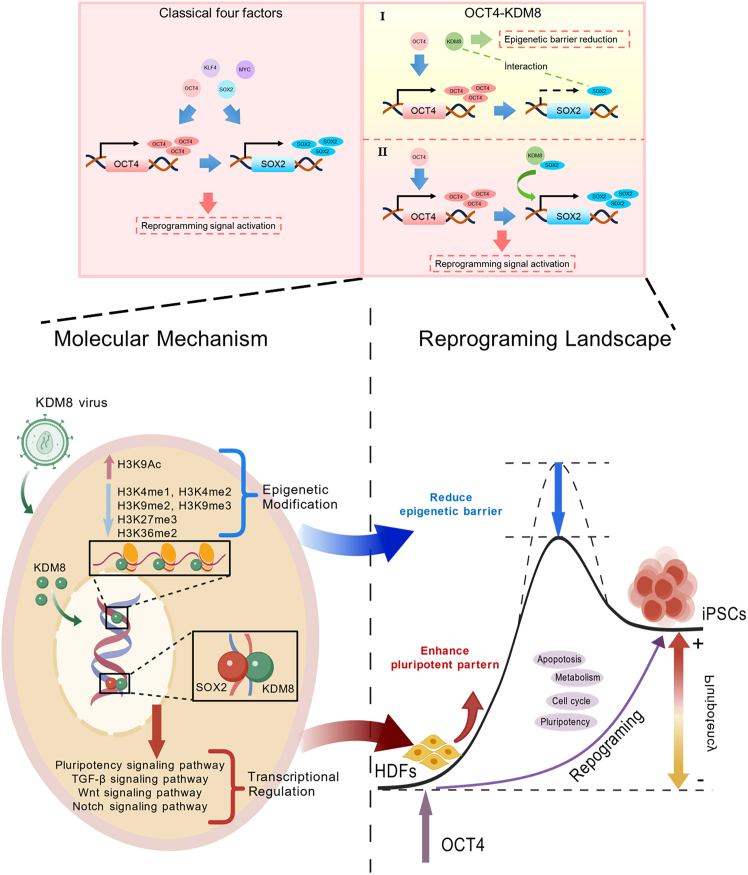
KDM8 facilitates reprogramming through dual regulatory modes KDM8 functions as both a transcriptional co-regulator of SOX2 and an epigenetic modifier, promoting the formation of pluripotent stem cells by amplifying the pluripotency-associated transcriptional network while concurrently reducing the epigenetic barrier during reprogramming.

Building on the observed functional dichotomy, we supposed that *KDM8* facilitates reprogramming through dual regulatory modes. During cellular reprogramming, *OCT4* serves as a core pioneer factor that initiates the establishment of the pluripotency transcriptional network, yet fails to sustain complete reprogramming (Huyghe et al., 2024; King and Klose, 2017). In the OCT4-KDM8 reprogramming system, *OCT4* initiates low-level activation of endogenous *SOX2* expression (Figure S6B). Although this activation alone is insufficient to complete reprogramming (Figure S1B), it provides a substrate for *KDM8*. *KDM8* not only lowers the epigenetic barriers that need to be overcome during reprogramming but also, more importantly, it cooperatively regulates SOX2-mediated binding and transcriptional regulation of downstream gene networks. Consequently, the nascent pluripotency circuitry initiated by *OCT4* undergoes robust amplification and stabilization via *KDM8*-dependent mechanisms, enabling cells to progress efficiently along the reprogramming trajectory. Based on these findings, we propose a schematic model of the reprogramming landscape orchestrated by *KDM8* (Figure 7).

Collectively, our study demonstrates the potent reprogramming capacity of *KDM8*. It functions as a highly effective reprogramming factor that significantly enhances reprogramming efficiency while reducing the required number of exogenous transcription factors, thereby improving genomic stability and enhancing the potential use for tissue engineering. Furthermore, we reveal that KDM8 engages in transcriptional regulation through its interaction with SOX2. This finding unveils a novel facet of *KDM8* function beyond its canonical enzymatic activity, opening new avenues for future research into its biological roles.

## Resource availability

### Lead contact

Requests for further information and resources should be directed to and will be fulfilled by the lead contact, Jin Wang (jin.wang.1@stonybrook.edu).

### Materials availability

All the materials generated and used in this study will be available upon reasonable request.

### Data and code availability

The RNA-seq data have been deposited in the Gene Expression Omnibus (GEO) under accession numbers GSE324436 and GSE324498, and the ChIP-seq data under accession number GSE324499. These datasets will be publicly available upon publication.

## Acknowledgments

Z.J. was supported by the Natural Science Foundation of Jilin Province (grant no. YDZJ202201ZYTS365). S.Y., Z.J., L.L., and E.W. acknowledges the support from the 10.13039/501100001809National Natural Science Foundation of China, (grant no. 91430217), (grant no. 21721003) and (grant no. 12234019) and the Ministry of Science and Technology (MOST) of the People's Republic of China (grant no. 2016YFA0203200).

## Author contributions

Z.J. and J.W. contributed to the experimental design and the conceptual interpretation; S.Y., Z.J., Z.Y., and L.L. carried out the experiments; S.Y. and Z.J. collected the data and performed the statistical analysis; S.Y., Z.J., E.W., and J.W. contributed to writing and revising the manuscript. All authors have read and approved the final manuscript.

## Declaration of interests

The authors declare no competing interests.

## STAR★Methods

### Key resources table

**Table:** 

REAGENT or RESOURCE	SOURCE	IDENTIFIER
**Antibodies**		

TRA-1-60(S) (TRA-1-60(S)) Mouse Monoclonal Antibody	Cell Signaling Technology	Cat# 4746, RRID:AB_2119059
TRA-1-81 (TRA-1-81) Mouse Monoclonal Antibody	Cell Signaling Technology	Cat# 4745, RRID:AB_2119060
SSEA4 (MC813) Mouse Monoclonal Antibody	Cell Signaling Technology	Cat# 4745, RRID:AB_2119060
Anti-Nanog antibody [EPR2027(2)]	Abcam	Cat# ab109250, RRID:AB_10863442
Anti-PAX6 antibody [EPR15858]	Abcam	Cat# ab195045, RRID:AB_2750924
Anti-NCAM1 antibody [EP2567Y]	Abcam	Cat# ab75813, RRID:AB_2632384
Anti-FOXA2 antibody [EPR4466]	Abcam	Cat# ab108422, RRID:AB_11157157
Anti-mouse IgG (H + L), F(ab')2 Fragment (Alexa Fluor® 555 Conjugate)	Cell Signaling Technology	Cat# 4409, RRID:AB_1904022
Goat Anti-Rabbit IgG H&L (Alexa Fluor® 568)	Abcam	Cat# ab175471, RRID:AB_2576207
JMJD5 Polyclonal Antibody	Thermo Fisher Scientifi	Cat# PA5-44862, RRID:AB_2606711
Sox2 (D9B8N) Rabbit Monoclonal Antibody	Cell Signaling Technology	Cat# 23064, RRID:AB_2714146
Normal Rabbit IgG	Cell Signaling Technology	Cat# 2729, RRID:AB_1031062
JMJD5 Antibody (D-5)	Santa Cruz Biotechnology	Cat# sc-377078, RRID: AB_3751174
Anti-Histone H3 (acetyl K9) antibody [EPR16988] - ChIP Grade	Abcam	Cat# ab177177, RRID: AB_3750754
Anti-Histone H3 (di methyl K9) antibody [Y49] - ChIP Grade	Abcam	Cat# ab32521, RRID:AB_732927
Anti-Histone H3 (tri methyl K9) antibody [EPR16601] - ChIP Grade	Abcam	Cat# ab176916, RRID:AB_2797591
Anti-Histone H3 (mono methyl K4) antibody [ERP16597] - ChIP Grade	Abcam	Cat# ab176877, RRID:AB_2637011
Anti-Histone H3 (di methyl K4) antibody [EPR17707] - ChIP Grade	Abcam	Cat# ab176878, RRID: AB_3751173
Anti-Histone H3 (tri methyl K27) antibody [EPR18607] - ChIP Grade	Abcam	Cat# ab192985, RRID:AB_2650559
Anti-Histone H3 (di methyl K36) antibody [EPR16994(2)] - ChIP Grade	Abcam	Cat# ab176921, RRID:AB_2941920
β-actin antibody	X-blot	Cat# X52101, RRID: AB_3751175
β-actin antibody	Abmart	Cat# P30002, RRID:AB_2936505
Oct-4A (C30A3) Rabbit Monoclonal Antibody	Cell Signaling Technology	Cat# 2840, RRID:AB_2167691
Sox2 (D6D9) Rabbit Monoclonal Antibody	Cell Signaling Technology	Cat# 3579, RRID:AB_2195767
Nanog (D73G4) Rabbit Monoclonal Antibody	Cell Signaling Technology	Cat# 4903, RRID:AB_10559205
Goat Anti-Rabbit IgG (H + L)	ZSGB-Bio	Cat#ZB-2301, RRID: AB_2747412
Goat Anti-Mouse IgG (H + L)	ZSGB-Bio	Cat# ZB-2305, RRID: AB_2747415

**Biological samples**		

293T cells	National Collection of Authenticated Cell Cultures	Cat#GNHu44
Human dermal fibroblasts	MeisenCTCC	Cat#CTCC-197-HUM

**Chemicals, peptides, and recombinant proteins**		

ROCK inhibitor, Y27632	Selleck Chemicals	Cat# S1049
Camptothecin	MedChemExpress	Cat#HY-16560

**Critical commercial assays**		

BCIP/NBT Alkaline Phosphatase Color Development Kit	Beyotime Biotechnology	Cat#C3206
STEMdiff™ TrilLineage Differentiation Kit	STEMCELL Technologies	Cat#05230
Cell Counting Kit-8	Beyotime Biotechnology	Cat#C0042
FxCycle™ PI/RNase Staining Solution	Thermo Fisher Scientific	Cat#F10797
Annexin V-APC/7-AAD Apoptosis Detection Kit	Simu Biotechnology	Cat#A5001-03A-L
Seahorse XFp Glycolysis Stress Test Kit	Agilent Technologies	Cat#103017-100
Seahorse XFp Cell Mito Stress Test Kit	Agilent Technologies	Cat#103010-100
SimpleChIP Enzymatic Chromatin IP Kit	Cell Signaling Technology	Cat#9005

**Deposited data**		

RNA-Seq Raw and analyzed data 1	This paper	GEO: GSE324436
RNA-Seq Raw and analyzed data 2	This paper	GEO: GSE324498
ChIP-Seq Raw and analyzed data	This paper	GEO: GSE324499

**Experimental models: Organisms/strains**		

Mice:CB17/Icr-Prkdcscid/IcrlcoCrl	Charles River Laboratories	Cat#404

**Oligonucleotides**		

Primer for qPCR and ChIP-qPCR, see Table S7	This paper	Sequences listed in supplemental information

**Recombinant DNA**		

Lenti-EF1α-KDM8-IRES-EGFP	Youbio	Cat#VT1460
Lenti-EF1α-KDM8^E301-304^-IRES-EGFP	Youbio	Cat#VT1460
Lenti-EF1α-KDM8^CD^-IRES-EGFP	Youbio	Cat#VT1460
Lenti-EF1α-OCT4-IRES-EGFP	Youbio	Cat#VT1460
Lenti-EF1α-SOX2-IRES-EGFP	Youbio	Cat#VT1460
Lenti-EF1α-KLF4-IRES-EGFP	Youbio	Cat#VT1460
Lenti-EF1α-MYC-IRES-EGFP	Youbio	Cat#VT1460
Lenti-EF1α-shSOX2-IRES-EGFP	GeneChem	N/A
Lenti-EF1α-shKDM8-IRES-EGFP	GeneChem	N/A

**Software and algorithms**		

ImageJ	N/A	https://imagej.nih.gov/ij/
BioGDP.com	Jiang et al. (2025)	BioGDP - Generic Diagramming Platform for Biomedical Graphics
GraphPad	N/A	https://www.graphpad.com/features

### Experimental model and study participant details

#### Mice

Animal experiments were performed by WISH Biotechnology (Authorization Certificates: 20211020-02 and 20211022-01) under the institutional license SYXK 2019-0007 issued by the Department of Science and Technology of Jilin Province. A total of sixteen 4-week-old female CB-17 SCID mice (Charles River Laboratories, China) were used for teratoma assays (12 for experiment 20211020-02; 4 for 20211022-01). All animals were housed under specific pathogen-free conditions in accordance with the national standard GB14925, with free access to food and water.

#### Cell culture

All cells are cultured under the conditions of 37°C and 5% CO_2_ supplementation. 293T cells (National Collection of Authenticated Cell Cultures, China) are cultured in high-glucose DMEM medium (Gbico, USA) containing 10% fetal bovine serum (FBS) (Vivacell, China). Human dermal fibroblasts (HDFs) are purchased from MeisenCTCC (China) (CTCC-197-HUM), including STR profiling and certification confirming the absence of contamination. HDFs are cultured in the fibroblast culture medium provided by the company. The cells were passaged at a 1:2 ratio when they reached 90% confluency. The cells were cryopreserved in a solution of 90% FBS and 10% DMSO, stored in liquid nitrogen, and thawed in a 37°C water bath. HDFs were expanded for up to 9 passages. All experimental analyses were performed on cells within passages 4 to 6.

Induced pluripotent stem cells (iPSCs) were cultured in 6-well plates coated with hESC-qualified Matrix (Corning, USA), using TeSR-E8 (STEMCELL Technologies, Canada) medium, and the TeSR-E8 medium was replaced daily. Every 4–5 days, iPSCs were transferred to a new culture plate. When passaging iPSCs, wash the iPSCs with calcium and magnesium-free DPBS, and then add the Gentle Cell Dissociation Reagent (STEMCELL Technologies, Canada) to the culture dish. Incubate at room temperature for 5–8 min and observe under a microscope that the edges of most colonies detach from the bottom of the culture dish. Aspirate the digestion solution and immediately add fresh complete medium. Use a pipette to gently blow the bottom of the dish to detach the stem cell colonies attached to the bottom. Blow gently and slowly, mix, and transfer 1/6 of the cell solution to a new culture dish. Place the cells at room temperature for 30 min, and then culture them in a 37°C incubator containing 5% CO_2_.

### Method details

#### Lentiviral vector construction and iPSCs generation

The lentiviral vector *Lenti-EF1α-cDNA-IRES-EGFP*, encoding customizable expression of either wild-type KDM8 (UniProt ID: Q8N371), its mutants (KDM8^E301-304^ or KDM8^CD^) or individual reprogramming factors (OCT4, SOX2, KLF4, or MYC), was procured from Youbio (China). The sequences of shRNA were as follows: shSOX2 (GCTCTTGGCTCCATGGGTT), shKDM8 (AGGTACACAGATGAGGAATGG) (GeneChem, China). For lentiviral production, 293T cells cultured in 10 cm dishes were co-transfected using Lipofectamine 3000 transfection reagent (Invitrogen, USA) with the Lentiviral vector (5.8 μg) combined with pMD2.G (2.9 μg) and psPAX2 (5.8 μg). Following transfection, cells were maintained at 37°C in a 5% CO2 atmosphere for 8 h before medium replacement. Viral supernatants were harvested 36 h post-transfection and sequentially filtered through 0.22 μm Millex-HV syringe filters (Millipore, USA). The purified viral particles were aliquoted and cryopreserved at −80°C for subsequent transduction experiments.

Following titer determination for each lentiviral preparation (Table S6), HDFs were transduced with lentiviral vector mixtures carrying distinct transcription factor combinations. At 72 h post-transduction, transduced cells were quantified and replated at a density of 1 × 10^5^ cells per well in 6-well cell culture plates pre-coated with hESC-qualified Matrigel matrix (354277, Corning, USA). The culture medium was aspirated 24 h later and replaced with TeSR-E8 medium (STEMCELL Technologies, Canada). Daily medium replacement was performed until the emergence of human embryonic stem cell-like colonies. On day 30 post-lentiviral infection, colonies exhibiting characteristic iPSCs morphology were manually dissected and subsequently expanded in TeSR-E8 culture medium under standardized conditions.

#### Alkaline phosphatase (AP) staining and immunofluorescence analysis

To detect AP activity, cells were washed thrice with PBS and fixed with 4% paraformaldehyde for 10 min at room temperature. Following three TBST (PBS containing 0.1% Tween 20) washes, alkaline phosphatase activity was visualized using BCIP/NBT Alkaline Phosphatase Color Development Kit (Beyotime Biotechnology, China) according to the manufacturer’s protocol. Chromogenic reactions proceeded for 30 min at room temperature under light-protected conditions.

For immunofluorescence staining, cells were washed three times with PBS and fixed with 4% paraformaldehyde for 15 min at room temperature. Nuclear antigen detection (PAX6, NCAM1 and FOXA2) required permeabilization with PBS containing 2% Triton X-100 for 10 min, whereas membrane-associated antigens (SSEA4, TRA-1-60 and TRA-1-81) were processed without permeabilization. Non-specific binding was blocked with 1% bovine serum albumin (BSA) and 22.52 mg/mL glycine in PBS-T (PBS with 0.1% Tween 20) for 30 min. Primary antibodies were diluted in PBS-T containing 1% BSA and incubated overnight at 4°C. The antibodies used were as follows: TRA-1-60 (4746, 1:1000, Cell Signaling Technology, USA), TRA-1-81 (4745, 1:1000, Cell Signaling Technology, USA), SSEA4 (4755, 1:500, Cell Signaling Technology, USA), NANOG (ab109250, 1:200, Abcam, UK), PAX6 (ab195045, 1:350 Abcam, UK), NCAM1 (ab75813, 1:200 Abcam, UK) and FOXA2 (ab108422, 1:300 Abcam, UK). For secondary detection, the following antibodies were used: Alexa Fluor 555-conjugated Anti-Mouse IgG (4409, 1:1000, Cell Signaling Technology, USA) and Alexa Fluor 568-conjugated Anti-Rabbit IgG (ab175471, 1:1000, Abcam, UK). Nuclear counterstaining employed DAPI (Bioss, China) with imaging performed on an EVOS XL Core Imaging System (Thermo Fisher Scientific, USA).

#### Teratoma formation and cytogenetic analysis

Experimentally derived iPSCs (5 × 10^6^ cells/mouse) were subcutaneously injected into female CB-17 SCID mice (Charles River Laboratories, China). Teratomas developed within 8 weeks post-injection and were subsequently excised for histopathological processing. Resected tissues underwent paraffin-embedding followed by sectioning for hematoxylin and eosin (H&E) staining and immunohistochemical analysis. Histological specimens were imaged using a bright-field microscope (Nikon Eclipse Ti2, Nikon Instruments, Tokyo, Japan). A total of 16 mice were subcutaneously injected with pluripotent stem cells, with teratoma formation observed in all 16 cases (100% efficiency).

Cytogenetic profiling was conducted following standardized high-resolution G-banding protocols at Celliver Biotechnology (China). Metaphase chromosome spreads prepared from experimental samples were subjected to Giemsa staining and analyzed at 400-550-band resolution to assess chromosomal integrity and identify potential structural abnormalities.

#### In vitro trilayer lineage differentiation assay

iPSCs-O-KDM8 were subjected to directed differentiation using the STEMdiff TrilLineage Differentiation Kit (STEMCELL Technologies, Canada). Following enzymatic dissociation with Gentle Cell Dissociation Reagent (GCDR) to generate single-cell suspensions, cells were seeded into 12-well plates pre-conditioned with TeSR-E8 medium (STEMCELL Technologies, Canada) supplemented with 10 μM Y-27632 (ROCK inhibitor, Selleck Chemicals, USA). After 24-h stabilization, basal medium was replaced with lineage-specific differentiation media (Ectoderm, Mesoderm or Endoderm formulations). Daily medium replenishment was performed until termination at post-induction day 5 (mesoderm and endoderm) or 7 (ectoderm). Lineage commitment was validated through immunofluorescence detection of definitive markers: PAX6 (Ectoderm), NCAM1 (Mesoderm) and FOXA2 (Endoderm).

#### RNA extraction and RNA sequencing

Total RNA was isolated using the UNIQ-10 Silica-Matrix RNA Purification System (Sangon Biotech, China) following manufacturer-recommended protocols. Briefly, cellular lysates homogenized in Trizo Reagen underwent phase separation, followed by RNA purification via silica-membrane column chromatography. Sequential washes with Buffer PRE (70% ethanol formulation) effectively removed contaminants, and RNA was eluted in nuclease-free DEPC-treated water.

All RNA samples were sequenced by DNBSEQ (BGI Tech, China). Inter-sample correlations were assessed using Pearson and Spearman correlation coefficients. Each group consisted of three biological replicates: iPSCs samples derived from independent clones, while HDFs controls were triplicate samples from the same batch prior to induction. The heatmap was plotted by ChiPlot.

#### Quantitative polymerase chain reaction (qPCR)

First-strand cDNA synthesis was performed using the PrimeScript RT Master Mix (Takara, Japan). Gene expression profiling was conducted using TB Green Premix Ex Taq II (Takara, Japan). GAPDH was served as endogenous control. Relative quantification was calculated using the comparative Ct method (2^−ΔΔCt^) with three technical replicates. All qPCR primers are listed in Table S7.

#### Cell proliferation analysis via CCK-8 assay

Cellular proliferation kinetics of HDFs were quantified using the Cell Counting Kit-8 (CCK-8, Beyotime Biotechnology, China). Cells were seeded in 96-well plates at a density of 1×10^3^ cells/well with triplicate technical replicates per experimental group. At designated time points (days 1, 3, 5, 7, 9, 11, 13 and 15 post-seeding), 10 μL CCK-8 reagent was added to each well followed by 2-h incubation at 37°C under 5% CO_2_. Absorbance measurements were acquired at 450 nm using a Synergy H1 Hybrid Multi-Mode Reader (Agilent Technologies, USA). Data represent mean ± standard deviation (SD) from three independent biological replicates.

#### Cell cycle profiling via flow cytometry

HDFs were transduced with lentiviral constructs encoding KDM8, KDM8-targeting shRNA, or non-targeting lentiviral vector. Upon achieving 80% confluency, cells underwent trypsinization and centrifugation. Pelleted cells were washed twice with ice-cold PBS and fixed in 70% ice-cold ethanol at 4°C for 24 h. Fixed cells were rehydrated in PBS, treated with FxCycle PI/RNase Staining Solution (Thermo Fisher Scientific, USA) in darkness for 20 min at room temperature. After incubation, HDFs were subjected to flow cytometry.

#### Apoptosis analysis via flow cytometry

After 24 h of 40 μM camptothecin (HY-16560, MedChemExpress, USA) treatment to induce cell apoptosis, apoptosis quantification was performed using the Annexin V-APC/7-AAD Apoptosis Detection Kit (Simu Biotechnology, China) according to manufacturer specifications. Lentivirus-transduced HDFs (1×10^5^ cells/sample) were resuspended in 100 μL binding buffer containing 5 μL Annexin V-APC and incubated for 10 min at 25°C under light-protected conditions. Subsequent 7-AAD counterstaining (5 μL/sample) was conducted for 5 min. Samples were analyzed within 1 h using an Attune NxT Flow Cytometer (Thermo Fisher Scientific, USA).

#### Glycolytic and mitochondrial metabolic profiling via Seahorse XF technolog

Glycolytic metabolic flux was measured on an Agilent Seahorse XFp metabolic flux analyzer using a Glycolytic Stress test kit (Agilent Technologies, USA). For the measurement of extracellular acidification rate (ECAR), cells were successively treated with glucose, oligomycin and 2-DG. Data analysis was performed by Agilent Seahorse XF Glycolytic Rate Assay Report Generator. Mitochondrial respiratory flux was measured on an Agilent Seahorse XFp metabolic flux analyzer using a Cell Mito Stress Test kit (Agilent Technologies, USA). For the measurement of oxygen consumption rate (OCR), cells were successively treated with oligomycin, carbonyl cyanide-4-(trifluoromethoxy)phenylhydrazone (FCCP) and a mixture of rotenone and antimycin A. Data analysis was performed using the Agilent Seahorse XF Cell Mito Stress Test Report Generator.

#### Chromatin immunoprecipitation (ChIP) sequencing and ChIP-qPCR

HDFs (6×10^5^ cells) were seeded onto a 10-cm dish and infected with the indicated Lentivirus. Cells were cross-linked with 1% formaldehyde (Sigma, USA) for 10 min at room temperature and quenched with 0.125 M of glycine for 5 min at room temperature. Then the cells were washed three times with pre-iced PBS and centrifuged at 2,500 rpm for 5 min. Cell lysis, sonication and immunoprecipitation were performed using the SimpleChIP Enzymatic Chromatin IP Kit (Magnetic Beads) (9005, Cell Signaling Technology, USA) according to the manufacturer’s instructions. The antibodies for immunoprecipitation were KDM8 (PA5-44862, 2 μg/IP, Thermo Fisher Scientific, USA), SOX2 (23064, 10 μL/IP, Cell Signaling Technology, USA) and Normal Rabbit IgG (2729, 2 μg/IP, Cell Signaling Technology, USA). The ChIP product was sequenced by DNBSEQ (BGI Genomics, China). All qPCR primers are listed in Table S7.

#### Co-immunoprecipitation and western blotting

Cellular lysates were prepared in ice-cold lysis buffer (Beyotime Biotechnology, China). Following 30-min incubation on ice, lysates were clarified by centrifugation at 12,000 rpm for 10 min at 4°C. Protein concentrations were determined using the BCA Protein Assay Kit (Beyotime Biotechnology, China) with bovine serum albumin standards. The antibodies for immunoprecipitation were KDM8 (PA5-44862, 2 μg/IP, Thermo Fisher Scientific, USA) and Normal Rabbit IgG (2729, 2 μg/IP, Cell Signaling Technology, USA). Protein A/G Magnetic is from MedChemExpress (HY-K0202, USA). An equal amount of protein from each sample was separated in a 12.5%SDS–PAGE gels and blotted onto PVDF membranes (Millipore, USA). The membranes were incubated with primary antibodies at 4°C overnight, followed by horseradish peroxidase (HRP)-conjugated goat anti-mouse/rabbit IgG secondary antibody at room temperature for 40 min. Enhanced chemiluminescence (ECL)reagent (Millipore, USA) was used for detection. The membranes were incubated with EC, and proteins were visualized using DNR Bio-Imaging Systems. The grayscale intensities of the results were analyzed using ImageJ analytical software. Primary antibodies included: KDM8 (sc-377078, 1:100, Santa Cruz Biotechnology, USA), H3K9ac (ab177177, 1:1000, Abcam, UK), H3k9me2 (ab32521, 1:1000, Abcam, UK), H3K9me3 (ab176916, 1:1000, Abcam, UK), H3K4me1 (ab176877, 1:1000, Abcam, UK), H3K4me2 (ab176878, 1:1000, Abcam, UK), H3K27me3 (ab192985, 1:1000, Abcam, UK), H3K36me2 (ab176921, 1:1000, Abcam, UK), β-actin (X52101s, 1:2500, X-blot, China), β-actin (P30002, 1:2500, Abmart, China), OCT4 (2840, 1:1000, Cell Signaling Technology, USA), SOX2 (3579, 1:1000, Cell Signaling Technology, USA), NANOG (4903, 1:2000, Cell Signaling Technology, USA). For secondary detection, the following antibodies were used: Goat Anti-Rabbit IgG (H + L) (ZB-2301, 1:2500, Zhongshan Golden Bridge Biotechnology, China) and Goat Anti-Mouse IgG (H + L) (ZB-2305, 1:2500, Zhongshan Golden Bridge Biotechnology, China).

### Quantification and statistical analysis

Data are presented as the means ± standard deviations of three independent experiments. Independent-sample t tests were used for comparisons between two groups, and one-way ANOVA was used for comparisons among multiple groups. Differences with *p* values of less than 0.05 were considered statistically significant. All statistical analyses were performed using GraphPad Prism 10. Effect sizes for each analysis or graph are reported in the corresponding figure legends for transparency. Figure 7 was created with BioGDP.com (Jiang et al., 2025).

## Supplemental information

Document S1. Figures S1–S7, Tables S3, S6, and S7

Table S1. RNA-seq data of HDFs-KDM8 and HDFs-EGFP at day 5

Table S2. CoIP-MS detected proteins binding to KDM8 in HDFs-O*-*KDM8, related to Figure 5

Table S4. Genes not annotated as SOX2 targets identified in KDM8 ChIP-seq in HDFs-O-KDM8

Table S5. RNA-seq data of HDFs-O-KDM8 and HDFs-O-EGFP at day 5

Document S2. Article plus supplemental information
