# Supplementary material for: KDM8 acts as a co-regulator of transcription factor SOX2 for promoting cell pluripotency
Source: Stem Cell Reports. 2026 Jun 18;21(7):102963. doi: 10.1016/j.stemcr.2026.102963 (PMC13385443; doi:10.1016/j.stemcr.2026.102963)
Supplement: Document S1. Figures S1–S7, Tables S3, S6, and S7 [file mmc1.pdf]

Stem Cell Reports, Volume *21*

Supplemental Information

KDM8 acts as a co-regulator of transcription factor SOX2 for promoting cell pluripotency

Songqin Yang, Zhikai Ye, Lu Lin, Zhenlong Jiang, Erkang Wang, and Jin Wang

SUPPLEMENTAL INFORMATION

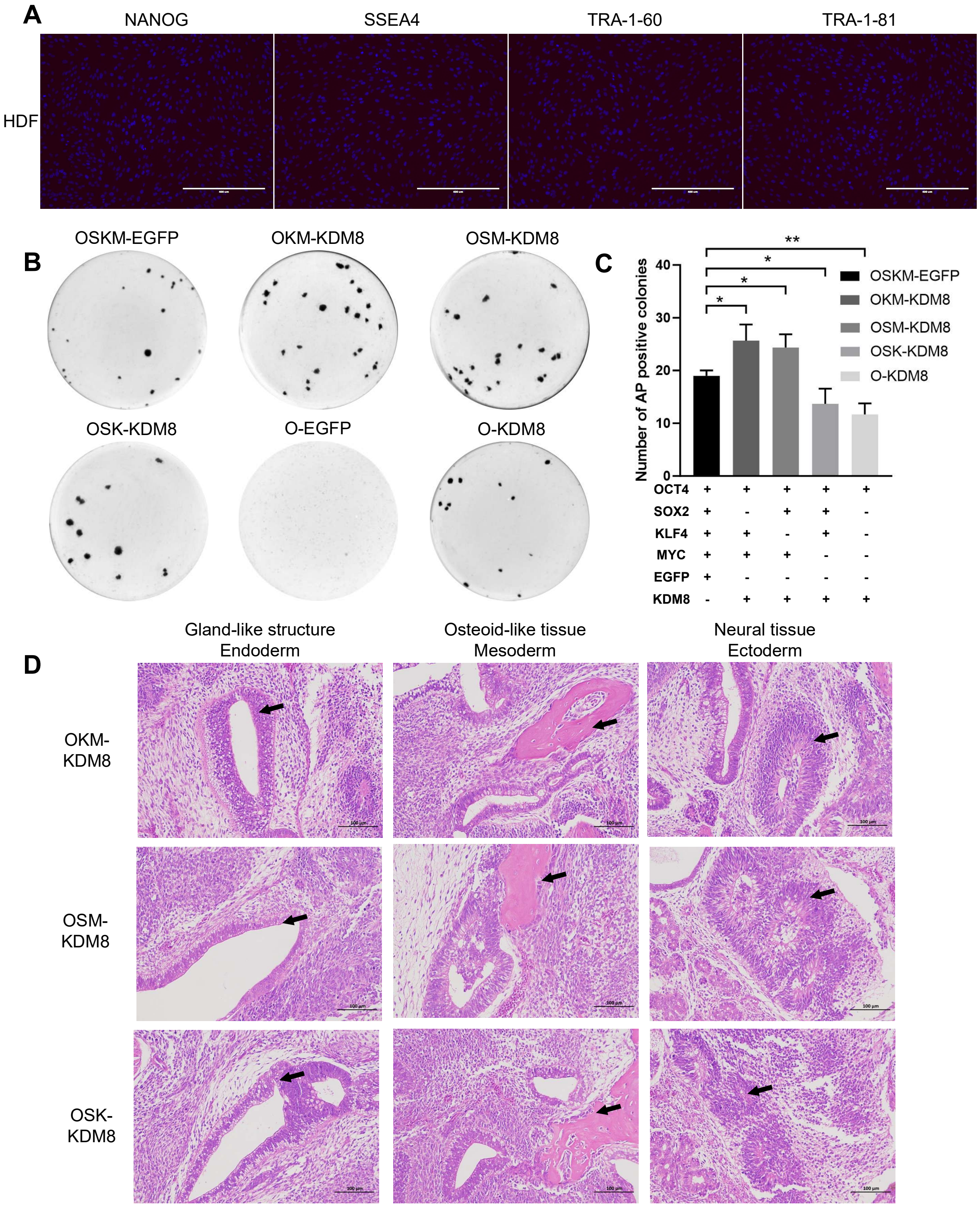

Figure S1. KDM8 can respectively replace OCT4, SOX2 and MYC, related to Figures 1 and 2.

(A) Negative immunofluorescence staining for pluripotency markers (NANOG, SSEA4, TRA-1-60, TRA-1-81) in HDFs. Representative merged images with DAPI nuclear staining are shown. Scale bar, 400 μm.

(B) and (C) Alkaline phosphatase positive clones of OSKM-EGFP, OKM-KDM8, OSM-KDM8, OSK-

KDM8, O-EGFP and O-KDM8 induced HDFs into iPSCs at day 30. No AP-positive clones were detected for O-EGFP. Data are represented as the mean ± SD, n = 3 independent experiments. *$P < 0.05$.

(D) H&E staining of teratomas developed by injecting iPSCs-OKM-KDM8, iPSCs-OSM-KDM8 and iPSCs-OSK-KDM8 into CB-17 SCID mice, which revealed three germ layers (endoderm, mesoderm and ectoderm) (Scale bar, 100 μm).

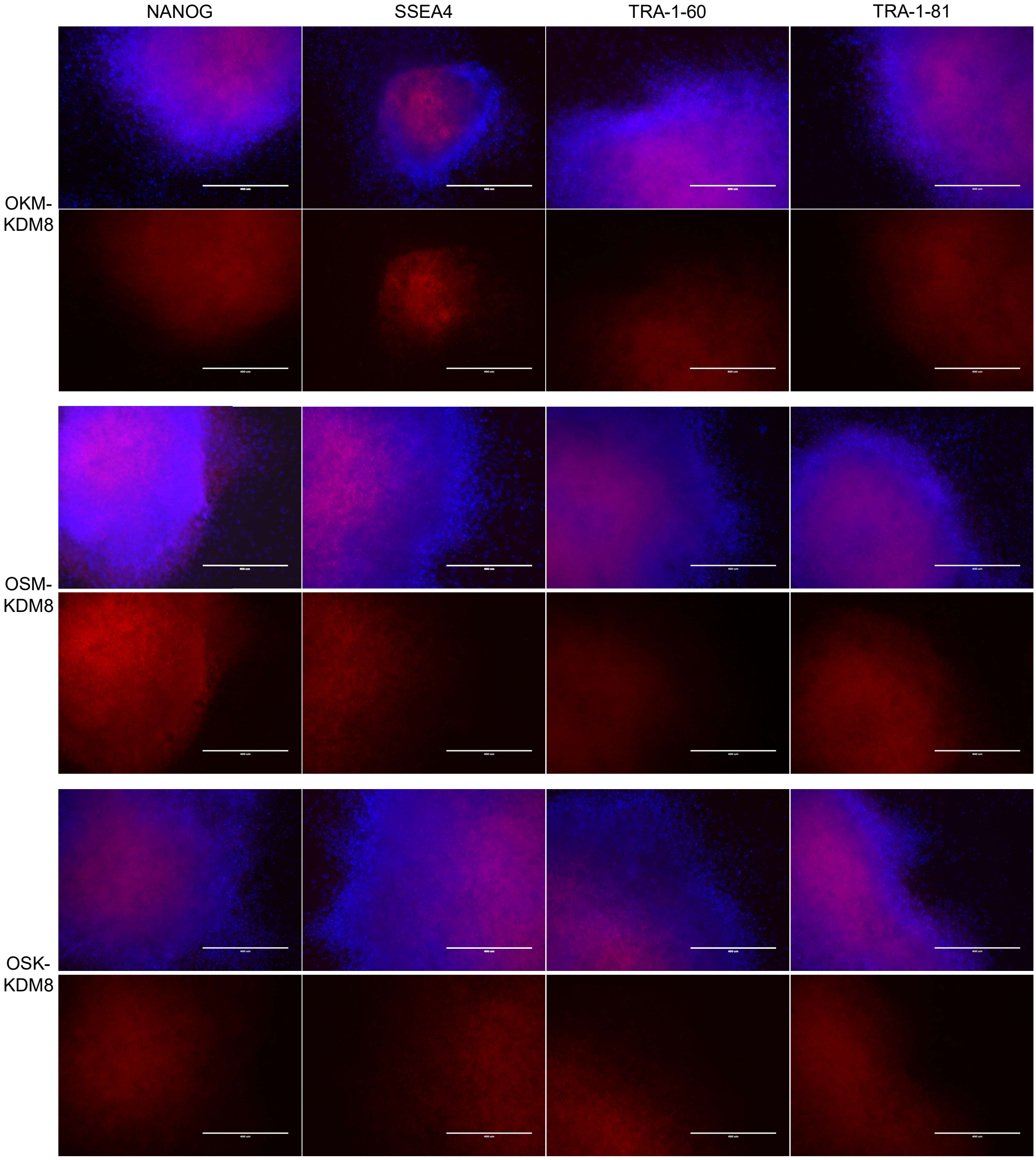

Figure S2. KDM8 can respectively replace OCT4, SOX2 and MYC, related to Figure 2. Immunofluorescence staining of pluripotency markers (NANOG, SSEA4, TRA-1-60, TRA-1-81) in iPSCs-OKM-KDM8, iPSCs-OSM-KDM8, and iPSCs-OSK-KDM8. Single-channel and merged images with DAPI nuclear counterstain are shown. Scale bar, 400 μm.

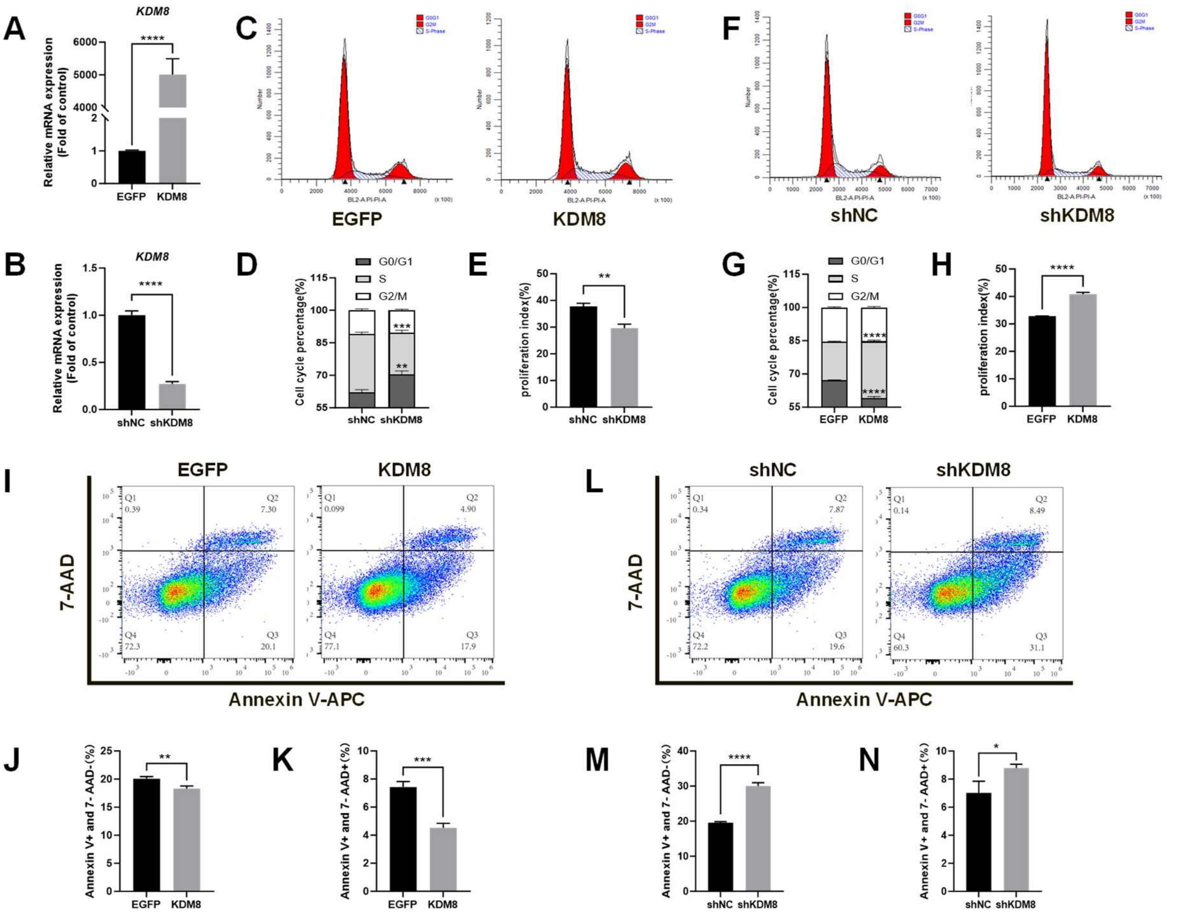

Figure S3. KDM8 can promote the cell cycle and resist cell apoptosis, related to Figure 3.

(A) and (B) KDM8 expression in HDFs overexpressing KDM8 or shKDM8 was determined by qPCR at day 3. Data are represented as the mean ± SD, n = 3 independent experiments. ****P < 0.0001.

(C) Effects of KDM8 in the cell cycle distribution in HDFs at day 5 post-transfection. n = 3 independent experiments.

(D) Percentages of cells in the G1, S and G2 phases of the cell cycle and PIs (E) of HDFs-KDM8 and HDFs-EGFP. Data are represented as the mean ± SD, n = 3 independent experiments. **P < 0.01; ***P < 0.001.

(F) Effects of shKDM8 in the cell cycle distribution in HDFs at day 5 post-transfection. n = 3 independent experiments.

(G) Percentages of cells in the G1, S and G2 phases of the cell cycle and PIs (H) of HDFs-shKDM8 and HDFs-shNC. Data are represented as the mean ± SD, n = 3 independent experiments. ****P < 0.0001.

(I) Flow cytometric quantification of apoptotic populations in HDFs-KDM8 vs HDFs-EGFP at day 5: early apoptotic cells (APC Annexin V+ and 7-AAD-) (J) and late apoptotic cells (APC Annexin V+ and 7-AAD+) (K). Data are represented as the mean ± SD, n = 3 independent experiments. **P < 0.01; ***P < 0.001.

(L) Flow cytometric quantification of apoptotic populations in HDFs-shKDM8 vs HDFs-shNC at day 5: early apoptotic cells (APC Annexin V+ and 7-AAD-) (M) and late apoptotic cells (APC Annexin V+ and 7-AAD+) (N). Data are represented as the mean ± SD, n = 3 independent experiments. *P < 0.05; ****P < 0.0001.

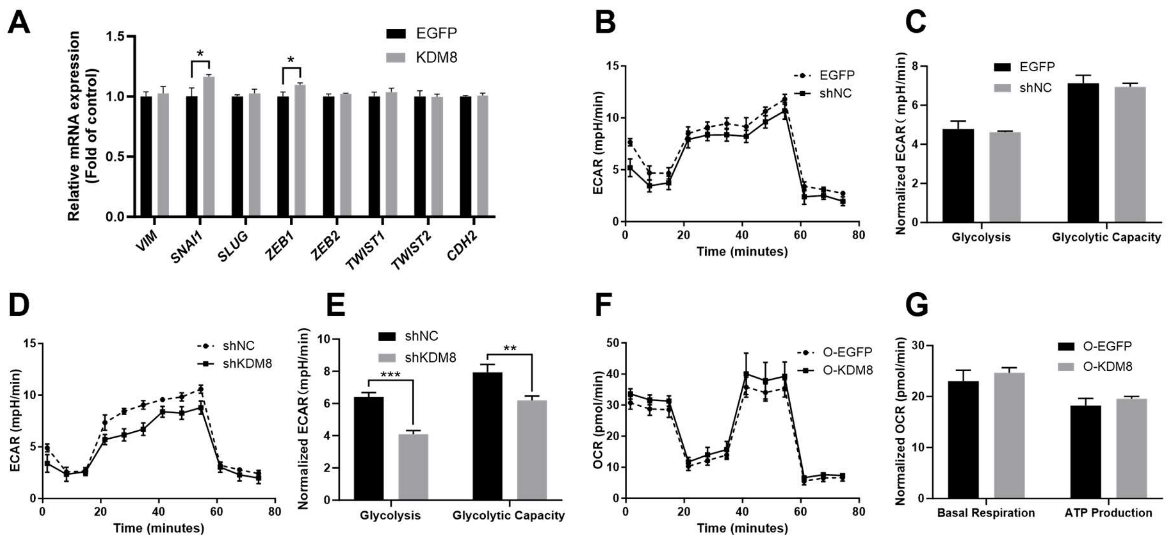

Figure S4. KDM8 promotes the TGF-β signaling pathway and glycolytic metabolism, related to Figure 4.

(A) Expression of TGF-β signaling pathway related genes in HDFs-KDM8 and HDFs-EGFP at day 15 was assessed by qPCR. Data are represented as the mean ± SD, n = 3 independent experiments. *P < 0.05.

(B) and (C) Glycolysis function of HDFs expressing EGFP and shNC at day 5. Data are represented as the mean ± SD, n = 3 independent experiments.

(D) and (E) Glycolysis function of HDFs expressing shKDM8 and shNC at day 5. Data are represented as the mean ± SD, n = 3 independent experiments. **P < 0.01; ***P < 0.001.

(F) and (G) Mitochondrial respiration in HDFs-O-KDM8 and HDFs-O-EGFP at day 5. Data are represented as the mean ± SD, n = 3 independent experiments. **P < 0.01; ***P < 0.001.

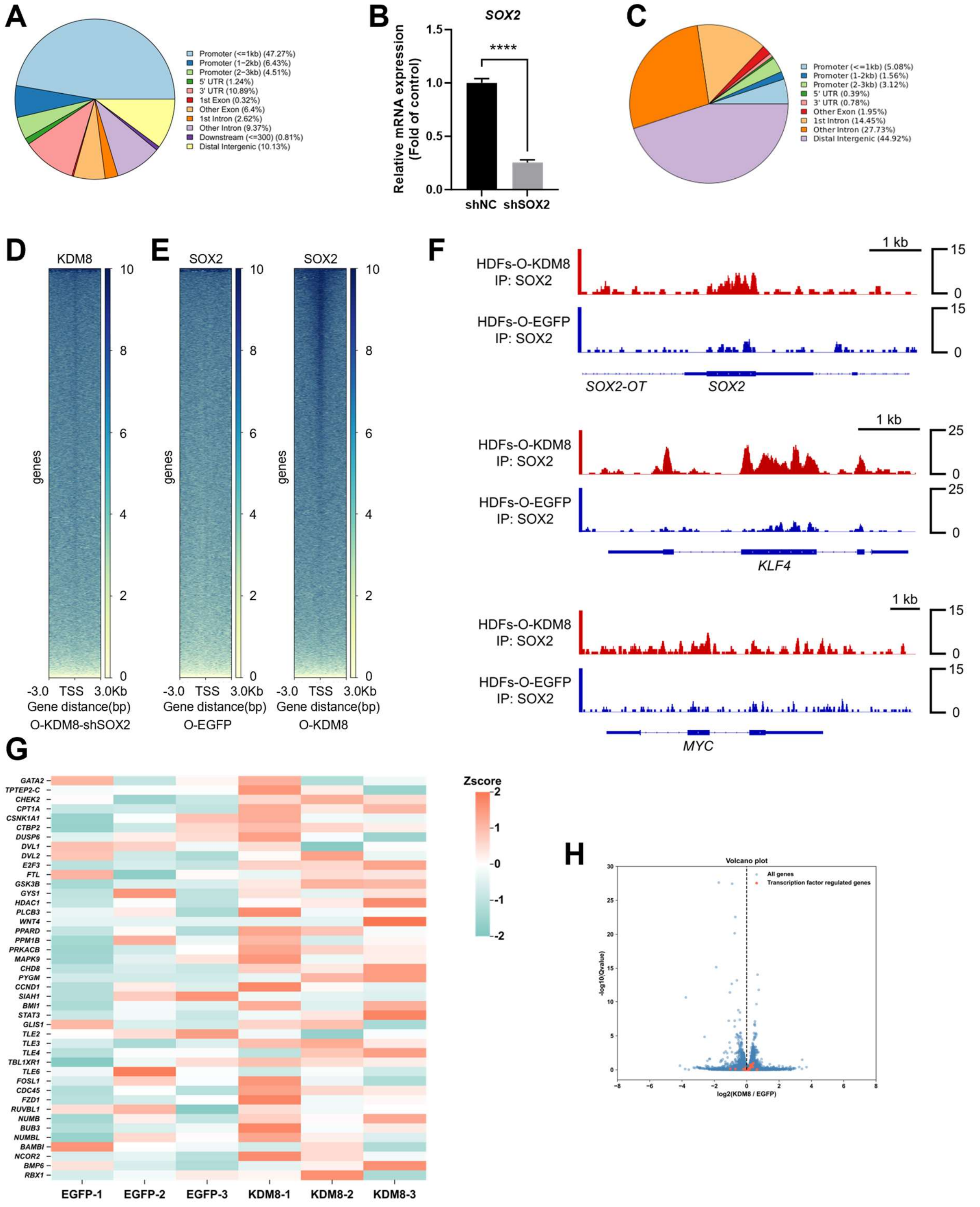

Figure S5. Related to Figure 5.

(A) Genomic distribution of KDM8 ChIP-seq peaks in HDFs-O-KDM8 at day 8.

(B) *SOX2* expression in HDFs overexpressing shSOX2 was determined by qPCR at day 3. Data are represented as the mean ± SD, n = 3 independent experiments. ****P < 0.0001.

(C) Genomic distribution of KDM8 ChIP-seq peaks in HDFs-O-KDM8-shSOX2 at day 8.

(D) A heatmap of KDM8 ChIP-seq occupancy around the TSS (± 3 kb) in HDFs-O-KDM8-shSOX2 at day 8.

(E) Heatmaps of SOX2 ChIP-seq occupancy around the TSS (± 3 kb) in HDFs-O-EGFP and HDFs-O-

KDM8 at day 8.

(F) Genome views of SOX2 tag density at *SOX2*, *KLF4* and *MYC* in HDFs-O-KDM8 and HDFs-O-EGFP.

(G) Heatmap of genes co-regulated by KDM8 and SOX2 plotted using RNA-seq data of HDFs-KDM8 vs HDFs-EGFP at day 5.

(H) Volcano plot of RNA-seq data (HDFs-KDM8 vs HDFs-EGFP, day 5). Genes presented in the heatmap (Figure S5G) are highlighted.

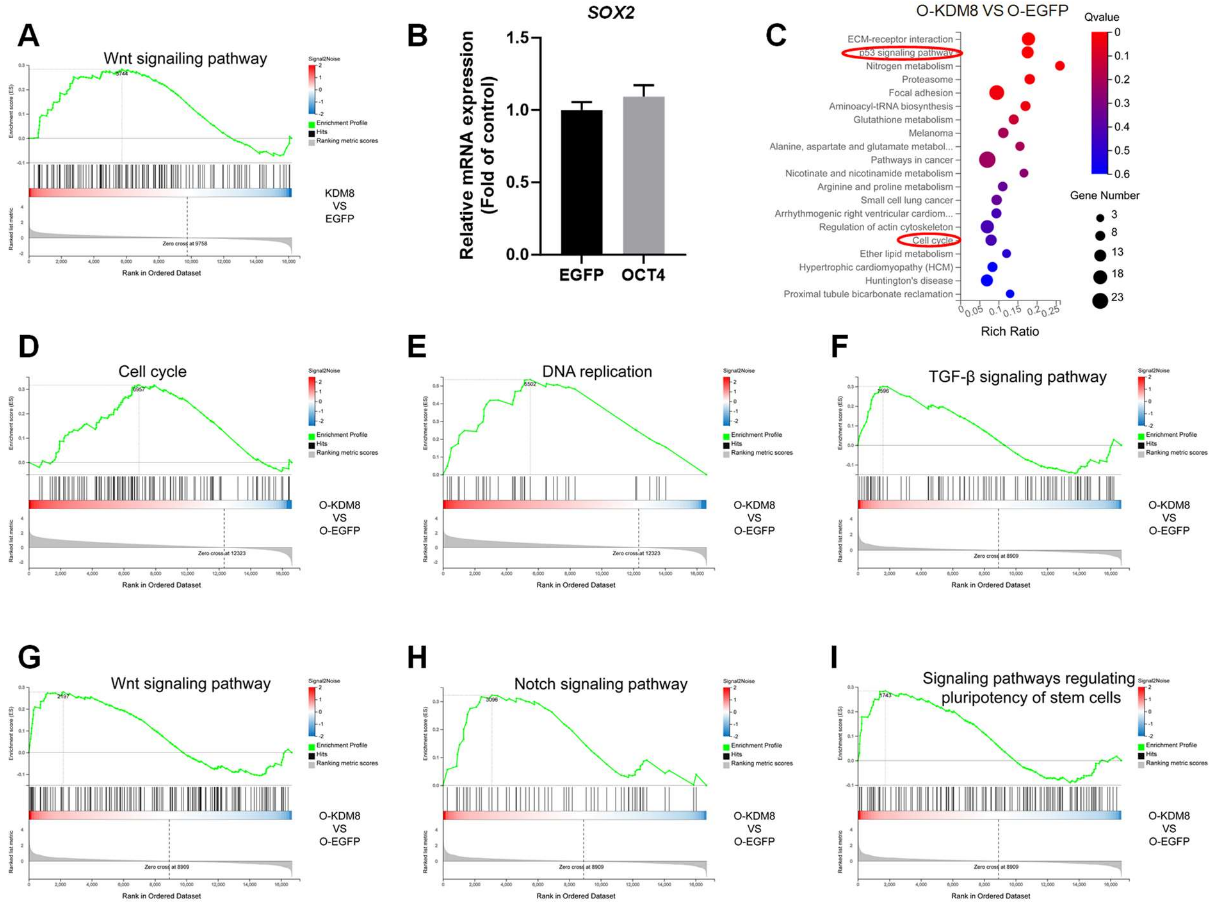

Figure S6. KDM8 can stabilize and amplify the pluripotency network activated by OCT4.

(A) GSEA of RNA-seq data from HDFs-KDM8 vs HDFs-EGFP at day 5: Reactome enrichment plots showed enrichment in Wnt signaling pathway.

(B) Compared to the EGFP group, HDF cells overexpressing OCT4 showed a slight increase in *SOX2* expression levels. n = 3 independent experiments.

(C) KEGG pathway analysis of RNA-seq data from HDFs-O-KDM8 vs HDFs-O-EGFP at day 5.

(D-I) GSEA of RNA-seq data from HDFs-O-KDM8 vs HDFs-O-EGFP at day 5: Reactome enrichment plots showed enrichment in the cell cycle, DNA replication, TGF-β signaling pathway, Wnt signaling pathway, Notch signaling pathway and signaling pathways regulating pluripotency of stem cells.

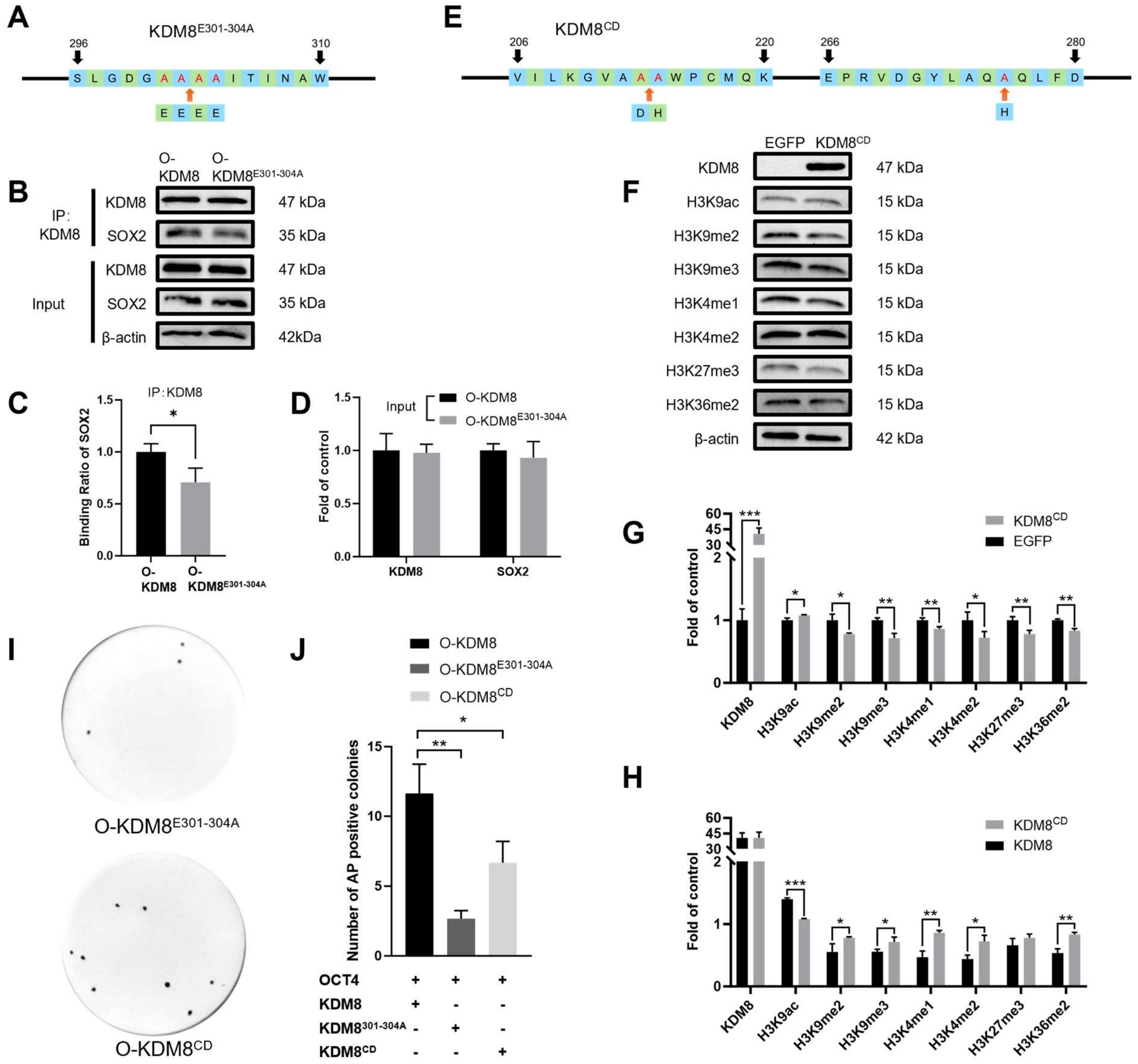

Figure S7. SOX2-binding or catalytic domain mutations in KDM8 impair reprogramming efficiency. Related to Figure 7.

(A) Schematic representation of SOX2-binding defective mutant (KDM8$^{E301\text{-}304A}$).

(B-D) Co-IP was employed to quantify the binding affinity between SOX2 and KDM8 in HDFs-O-KDM8 and HDFs-O-KDM8$^{E301\text{-}304A}$ at day 8**.** β-actin was used as an endogenous control for equal loading. Data are represented as the mean ± SD, n = 3 independent experiments. *P < 0.05.

(E) Schematic representation of Catalytically inactive mutant (KDM8^{CD}).

(F) and (G) Western blot analysis of the expression levels of KDM8, H3K9ac, H3K9me2, H3K9me3, H3K4me1, H3K4me2, H3K27me3 and H3K36me2 proteins in HDFs expressing EGFP and KDM8^{CD} at day 8. β-actin was used as an endogenous control for equal loading. Data are represented as the mean ± SD, n = 3 independent experiments. *P < 0.05; **P < 0.01; ***P < 0.001.

(H) Comparing of the expression levels of KDM8, H3K9ac, H3K9me2, H3K9me3, H3K4me1, H3K4me2, H3K27me3 and H3K36me2 proteins in HDFs expressing KDM8 and KDM8^{CD} at day 8.

(I) and (J) Alkaline phosphatase positive clones of O-KDM8, O-KDM8$^{E301\text{-}304A}$ and O-KDM8^{CD} induced HDFs into iPSCs at day 30. Data are represented as the mean ± SD, n = 3 independent experiments. **P < 0.01.

Table S3. SOX2 target genes identified in KDM8 ChIP-seq in HDFs-O-KDM8

P value	Gene ID	Symbol	Gene Start	Gene End
7.6531	347689	*SOX2-OT*	181056680	181742228
7.90374	9314	*KLF4*	107484852	107489769
6.74538	4609	*MYC*	127735434	127742951
5.58827	2624	*GATA2*	128479427	128488530
5.6562	102800317	*TPTEP2-CSNK1E*	38290691	38398915
10.4486	11200	*CHEK2*	28687743	28741820
4.86611	1374	*CPT1A*	68754620	68839684
6.89582	1452	*CSNK1A1*	149492982	149551439
4.8184	1488	*CTBP2*	124984317	125006018
4.81697	1848	*DUSP6*	89347235	89352501
5.55207	1855	*DVL1*	1335278	1349141
8.28056	1856	*DVL2*	7225341	7234544
5.55261	1871	*E2F3*	20401879	20493714
5.92313	2512	*FTL*	48965309	48966879
6.95288	2932	*GSK3B*	119996630	120095823
7.3731	2997	*GYS1*	48968130	48993309
7.86007	3065	*HDAC1*	32292083	32333626
7.07866	5331	*PLCB3*	64251530	64267923
5.96315	54361	*WNT4*	22117308	22142312
5.52825	5467	*PPARD*	35342558	35425400
9.20753	5495	*PPM1B*	44168853	44218518
4.59474	5567	*PRKACB*	84078079	84205437
18.6852	5601	*MAPK9*	180233143	180292083
12.319	57680	*CHD8*	21385199	21437275
6.25657	5837	*PYGM*	64746389	64760715
6.57604	595	*CCND1*	69641156	69654474
5.59912	6477	*SIAH1*	48354581	48384800
5.36844	100532731	*COMMD3-BMI1*	22316388	22331484
5.92347	6774	*STAT3*	42313324	42388442
5.77699	148979	*GLIS1*	53577214	53739171
7.25285	7089	*TLE2*	2997644	3047635
25.4422	7090	*TLE3*	70053229	70098171
9.69759	7091	*TLE4*	79571965	79726882
5.19618	79718	*TBL1XR1*	177019344	177197482
9.39311	79816	*TLE6*	2977410	2995184
5.75435	8061	*FOSL1*	65892049	65900388
4.95956	8318	*CDC45*	19479826	19520612
4.63941	8321	*FZD1*	91264433	91271326
5.34549	8607	*RUVBL1*	128064611	128123822
15.4698	8650	*NUMB*	73275216	73458546
9.1139	9184	*BUB3*	123154402	123165365
6.02322	9253	*NUMBL*	40665905	40690164
7.29936	25805	*BAMBI*	28677521	28682932

4.74669	9612	*NCOR2*	124324415	124567612
6.06883	654	*BMP6*	7726099	7881728
8.24286	9978	*RBX1*	40951378	40973309

Table S6. Lentivirus titer and MOI.

Lentivirus	Vital titers ($\times 10^8$)	MOI (TU/ml)
EGFP	2.10	20
OCT4	2.44	20
SOX2	1.28	20
KLF4	1.10	15
MYC	5.40	20
KDM8	2.61	20
shNC	2.08	15
shSOX2	2.23	15
shKDM8	2.15	15
$KDM8^{E301\text{-}304A}$	2.09	20
$KDM8^{CD}$	1.74	20

Table S7. Primers used for qPCR.

Gene symbol	Forward primers (5′ to 3′)	Revers primers (5′ to 3′)
For gene expression		
GAPDH	AGGGCTGCTTTTAACTCTGGT	CCCCACTTGATTTTGGAGGGA
KDM8	CACAGATGAGGAATGGTCCCAG	GCTGATGTCCTGCTTCAACTCC
TP53	ACCTATGGAAACTACTTCCTGAAA	CTGGCATTCTGGGAGCTTCA
CDKN1A	GATGGAACTTCGACTTTGTCAC	GTCCACATGGTCTTCCTCTG
CDKN2A	GGGTTTTCGTGGTTCACATCC	CTAGACGCTGGCTCCTCAGTA
CCND1	TCTACACCGACAACTCCATCCG	TCTGGCATTTTGGAGAGGAAGTG
CTNNB1	CACAAGCAGAGTGCTGAAGGTG	GATTCCTGAGAGTCCAAAGACAG
VIM	AGGCAAAGCAGGAGTCCACTGA	ATCTGGCGTTCCAGGGACTCAT
SNAI1	TGCCCTCAAGATGCACATCCGA	GGGACAGGAGAAGGGCTTCTC
SLUG	ATCTGCGGCAAGGCGTTTTCC	GAGCCCTCAGATTTGACCTGTC
ZEB1	GGCATACACCTACTCAACTACGG	TGGGCGGTGTAGAATCAGAGTC
ZEB2	AATGCACAGAGTGTGGCAAGGC	CTGCTGATGTGCGAACTGTAGG
TWIST1	GCCAGGTACATCGACTTCCTCT	TCCATCCTCCAGACCGAGAAGG
TWIST2	GCAAGATCCAGACGCTCAAGCT	ACACGGAGAAGGCGTAGCTGAG
CDH2	CCTCCAGAGTTTACTGCCATGAC	GTAGGATCTCCGCCACTGATTC
KLF4	CATCTCAAGGCACACCTGCGAA	TCGGTCGCATTTTTGGCACTGG
MYC	CCTGGTGCTCCATGAGGAGAC	CAGACTCTGACCTTTTGCCAGG
GLUT1	CAGTTTGGCTACAACACTGG	TGTAGAACTCCTCGATCACC
AKT1	TGGACTACCTGCACTCGGAGAA	GTGCCGCAAAAGGTCTTCATGG
PDK1	CATGTCACGCTGGGTAATGAGG	CTCAACACGAGGTCTTGGTGCA
HIF1A	TATGAGCCAGAAGAACTTTTAGGC	CACCTCTTTTGGCAAGCATCCTG
KRAS	CAGTAGACACAAAACAGGCTCAG	TGTCGGATCTCCCTCACCAATG
OCT4	GAGAAGGAGAAGCTAGAGCAAA	CTGTGTATATCCCAGGGTGATC
SOX2	GCTACAGCATGATGCAGGACCA	TCTGCGAGCTGGTCATGGAGTT
NANOG	CTCCAACATCCTGAACCTCAGC	CGTCACACCATTGCTATTCTTCG
CTCF	AAAGTGATTTGGGTGTCCAC	AACACAGCATCACAGTAACG
WDR5	ACTCAGAGCAAGCCTACAC	GCTGAATTTCACGGAGGAC
EZH2	GACCTCTGTCTTACTTGTGGAGC	CGTCAGATGGTGCCAGCAATAG
For ChIP qPCR		
SOX2	TGGTCGCTAGAAACCCATTT	TCTGCCTTGACAACTCCTGA
KLF4	GGCGCAGGTTCGGTCG	GCTGACCCCACCAGTCTTCG
MYC	TTTTGCCCTGCGTGACCA	CGTCTGCTTGAATGGACAGG
LIN28A	AGCCATATGGTAGCCTCATG	TCCCACTACTTCTCCCTCTG
JUNB	CCAGCTGCGCCTTCCTCAAA	TGGGTTTCTCTTCCGCTGTG
JUND	CCTGCAGCTCCAAAGCCACT	GTTGGGTTGGAGGTAGTGCG
BMI1	CCTGCAGCTCCAAAGCCACT	GTTGGGTTGGAGGTAGTGCG
GLIS1	CCTGCAGCTCCAAAGCCACT	GTTGGGTTGGAGGTAGTGCG
CCND1	TGCCGGGCTTTGATCTTT	CGGTCGTTGAGGAGGTTGG
CTNNB1	CCTGCAGCTCCAAAGCCACT	GTTGGGTTGGAGGTAGTGCG
GAPDH	CTCCTGTGGCATCCACGAAA	CGCCTGCTTCACCACCTTCT
